# Evaluation of lncRNA Expression Pattern and Potential Role in Heart Failure Pathology

**DOI:** 10.1155/2023/2369352

**Published:** 2023-07-12

**Authors:** Xiatian Chen, Chengzhen Shi, Jinning Gao, Juan Carlos Cueva Jumbo, Yin Wang, Xin Li, Cheng Zhao, Hua Yu, Peifeng Li, Lynn Htet Htet Aung

**Affiliations:** ^1^Institute of Translational Medicine, The Affiliated Hospital of Qingdao University, Qingdao University, Qingdao, China; ^2^School of Basic Medicine, Qingdao University, Qingdao, China; ^3^Juxian People's Hospital, Rizhao, China; ^4^School of Preclinical Medicine, Nanobody Research Center, Guangxi Medical University, Nanning, China; ^5^The Affiliated Cardiovascular Hospital of Qingdao University, Qingdao, China

## Abstract

During the last few decades, the morbidity and mortality of heart failure (HF) have remained on an upward trend. Despite the advances in therapeutic and diagnostic measures, there are still many aspects requiring further research. This study is aimed at finding potential long noncoding RNAs (lncRNAs) that could aid with the diagnosis and treatment of HF. We performed RNA sequencing on the peripheral blood of healthy controls as well as HF patients. The expression of lncRNAs was validated by RT-qPCR. Bioinformatic analysis was performed to investigate the possible mechanism of differentially expressed lncRNAs and mRNAs. The diagnostic value of lncRNAs was analysed by ROC analysis. Finally, a total of 207 mRNAs and 422 lncRNAs were identified. GO and KEGG pathway analyses revealed that biological pathways such as immune response, regulation of cell membrane, and transcriptional regulatory process were associated with the pathological progress of HF. The lncRNA-mRNA coexpression network was conducted, and several mRNAs were identified as key potential pathological targets, while lncRNA CHST11, MIR29B2CHG, CR381653.1, and FP236383.2 presented a potential diagnostic value for HF. These findings provide novel insights for the underlying mechanisms and possible therapeutic targets for HF.

## 1. Introduction

At present, statistics show that cardiovascular diseases (CVDs) are the leading cause of death around the world. Among them, it is estimated that about 40 million people suffer from heart failure (HF) in the whole world, and this number continues to grow due to the rapidly aging population as well as the global decline in birthrate [1, 2]. In addition, the aggravating changes in people's lifestyles, sedentarism and the high-sugar and high-fat diets, are starting to show an increase in the incidence of HF in the younger populations, particularly in developing countries. In older individuals, HF tends to occur at the final stage of a series of CVDs, such as in the case of myocardial infarction and hypertension [3]. Left ventricular ejection fraction (LVEF) has always been regarded as its pathological phenotype, but it cannot be widely used due to its inconvenience [4, 5]. Although there are novel and more effective measures to intervene in HF, the overall prognosis remains not optimistic [6, 7]. Therefore, a deeper understanding of the pathological mechanism behind HF is critical for effective diagnosis, treatment, and prognosis.

Long noncoding RNA (lncRNA) is a class of noncoding RNA which does not encode any proteins but plays a role in regulating mRNA transcription, protein location, and other cellular biological process [8, 9]. Previous studies revealed the role of dysregulated lncRNAs in the development and progression of CVDs [10–13]. Overexpression of the lncRNA cardiac physiological hypertrophy-associated regulator (CPhar) could prevent ischemia-reperfusion-induced apoptosis and cardiac dysfunction [14]. LncRNA noncoding repressor of nuclear factor of activated T cells (NRON) and myosin heavy-chain-associated RNA transcripts (MHRT) were both overexpressed in the plasma of HF patients [15]. The exploration of the correlation between lncRNAs and CVD has increased our understanding of CVD pathology and provided new therapeutic targets for CVD.

In this study, we performed RNA sequencing on the peripheral blood collected from 100 volunteers (50 HF patients and 50 healthy controls). RT-qPCR was used to identify the differentially expressed genes. Functional enrichment analysis was used on both the GO and KEGG databases. We conducted the lncRNA-mRNA coexpression network and reported several potential target genes by cis- and transregulation prediction. We then hypothesized that the mRNA-lncRNA expression profiles can provide important insights to understand the underlying mechanism of HF, and the differentially expressed lncRNAs may facilitate the finding of new diagnostic targets as well as aid drug development.

## 2. Materials and Methods

### 2.1. Subjects and Blood Sample

**Figure 1:**
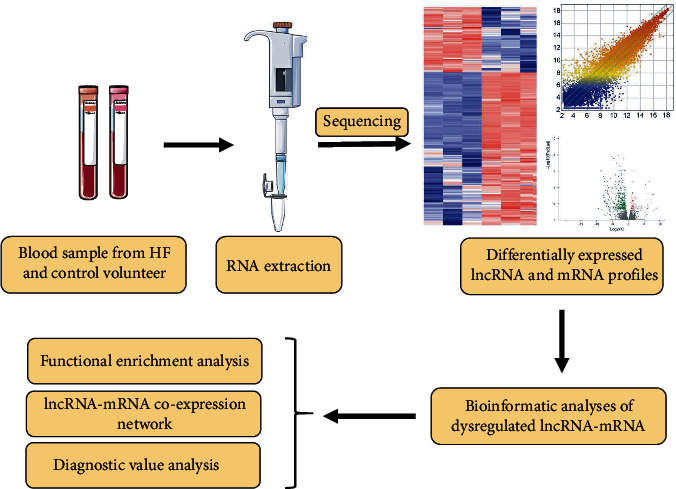
Research design flowchart. HF: heart failure; lncRNA: long noncoding RNA; mRNA: messenger RNA.

In the present study, peripheral whole blood samples were collected from a total of fifty HF patients and fifty normal controls at the Affiliated Hospital of Qingdao University between December 2018 and January 2020. The selection criteria for HF patients were as follows: (1) The patient must have been admitted to the hospital without any history of related disorders or treatments prior to being diagnosed; (2) NT-proBNP (N-teminal pro-B type natriuretic peptide)≥400 ng/L [16]; and (3) LVEF <50% [16, 17]. For the control group, we selected healthy individuals (in a similar age group) without a history of heart-related diseases and with normal echocardiograms. Among all participants, a total of four HF patients as well as healthy controls were selected for sequencing. This study was approved by the ethics committee of the Affiliated Hospital of Qingdao University. Each volunteer agreed and signed an informed consent form. The detailed workflow of this study is shown in Figure 1.

### 2.2. RNA Isolation

Total RNA was extracted from the blood samples using RNA isolate Total RNA Extraction Reagent (Vazyme, China) according to the manufacturer's instructions. In brief, 100 *μ*l of blood was combined with 400 *μ*l of RNA extraction reagent. After mixing, add a fifth volume of chloroform. The mix was shaken vigorously by hand for 15 s and incubated for 3 min at room temperature. Centrifuged the mix samples at 12000 g for 15 min at 4°C. Removed the aqueous phase of the sample and added the equal volume of 100% isopropanol, incubating for 10 min at room temperature. The mixed samples were centrifuged at 12000 g for 10 min at 4°C. And then, the RNA pellet was washed two times with 75% ethanol, and the dried RNA pellet was resuspended in RNase-free water. RNA quality was verified using a 2100 Bioanalyzer (Agilent Technologies, Santa Clara, CA, USA) and the ND-2000 (NanoDrop Technologies). RNA samples (OD260/280 = 1.8 − 2.2, OD260/230 ≥ 2.0, RIN ≥ 8, 28S : 18S ≥ 1.0, and total RNA > 10 *μ*g) were used to construct a sequencing library.

### 2.3. Library Preparation and Sequencing

The RNA-sequencing transcriptome library was prepared following the TruSeqTM stranded total RNA Kit from Illumina (San Diego, CA) using 5 *μ*g of total RNA. Ribosomal RNA (rRNA) depletion instead of poly(A) purification was performed by the Ribo-Zero Magnetic Kit and then fragmented by fragmentation buffer. Later, first-stranded cDNA was synthesized with random hexamer primers. Then, we removed the RNA template and synthesized a replacement strand, incorporating dUTP in place of dTTP to generate ds cDNA. The incorporation of dUTP quenched the second strand during amplification because the polymerase did not incorporate past this nucleotide. AMPure XP beads were used to separate the ds cDNA from the second-strand reaction mix. A single “A” nucleotide was added to the 3′ ends of these blunt fragments to prevent them from ligating to one another during the adapter ligation reaction. Lastly, multiple indexing adapters were ligated to the ends of the double-stranded cDNA. Libraries were size-selected for cDNA target fragments (200-300 bp) on 2% low-range ultra-agarose, followed by PCR amplification using Phusion DNA polymerase (NEB) for 15 PCR cycles. After quantification by TBS380, the paired-end RNA sequencing library was sequenced with the NovaSeq 6000 (2 × 150 bp read length). In addition, 3 *μ*g of total RNA was ligated with sequencing adapters with the TruseqTM Small RNA sample prep kit (San Diego, CA, USA). Subsequently, cDNA was synthesized by reverse transcription and amplified with 12 PCR cycles to produce libraries. After being quantified by TBS380, deep sequencing was performed by Shanghai Majorbio Bio-Pharm Biotechnology Co., Ltd. (Shanghai, China).

The raw paired-end reads were trimmed, and the quality was controlled by SeqPrep (https://github.com/jstjohn/SeqPrep) and Sickle (https://github.com/najoshi/sickle) using the default parameters. Then, the clean reads were separately aligned to the reference genome with orientation mode using HIASAT software (https://ccb.jhu.edu/software/hisat2/index.shtml) [18]. The mapped reads of each sample were assembled by StringTie (https://ccb.jhu.edu/software/stringtie/index.shtml?t=example) in a reference-based approach [19]. The sequencing depth ranged from 15 to 30 M; approximately 98% of reads achieve the sequencing quality score of Q20, and 95% of the reads have the sequencing quality score of Q30. The sequencing error rates of sequencing data were from 0.015 to 0.017 (Table S1).

### 2.4. Differential Expression Analysis and Functional Enrichment

To identify differential expression genes between two groups, the expression level of each transcript was calculated according to the transcripts per million reads (TPM) method. RSEM (http://deweylab.biostat.wisc.edu/rsem/) was used to quantify gene abundances. The differential expression analysis was performed using the DESeq2, DEGseq, and EdgeR software. Volcano plots and scatter diagrams showed the signal intensity of differentially expressed genes. Hierarchical clustering was performed to display the expression patterns of differentially expressed genes in each sample. For the function analysis, we performed the Gene Ontology (GO) and Kyoto Encyclopedia of Genes and Genomes (KEGG) to the profile of differently expressed genes. The GO analysis was composed of three parts, including biological process (BP), cellular component (CC), and molecular function (MF). All differently expressed genes were mapped to the GO and KEGG databases. *P* value <0.05 indicates that there is significant enrichment in the pathway.

### 2.5. Construction of Target Gene Prediction and Regulatory Network

The correlation between differentially expressed mRNAs and differentially expressed lncRNAs was evaluated using Pearson's correlation coefficient from matched mRNA and ncRNA expression profile data. We set Pearson's correlation coefficients greater than 0.9 as significant. The potential target genes of lncRNAs were predicted via cisregulation or transregulation patterns. Cisregulation prediction suggests that the function of lncRNA is related to the protein-coding genes adjacent to the coordinates, and lncRNA located upstream and downstream of coding proteins may intersect with promoters or other cisacting elements of coexpressed genes, thus regulating gene expression at the transcriptional or posttranscriptional level. We analysed and screened the protein-coding genes nearest 10 kb upstream or downstream of lncRNA transcription start site. Transregulation prediction was performed using the RNAplex (University of Vienna, Vienna, Austria) software to find the mRNAs that had complementary sequences to the lncRNAs [20]. The interaction network was built and visually displayed using Cytoscape software.

### 2.6. Quantitative Real-Time PCR (RT-qPCR)

1 *μ*g of the total RNA was converted to cDNA using the HiScript III RT SuperMix for qPCR kit (Catalog number R323, Vazyme, China) according to the manufacturer's instructions. The reaction was performed in 0.2 ml PCR tubes using thermocyclers. The expression of lncRNAs was detected by the Bio-Rad CFX96 system and analysed by the CFX Manager software. SYBR Green mix kit was used for progress tracking (Catalog number Q711, Vazyme, China). Briefly, reactions were composed of 1X ChamQ Universal SYBR qPCR mix, 200 nM forward primer, 200 nM reverse primer, 10 ng cDNA, and ddH_2_O (up to 20 *μ*l). The thermal cycling conditions were as follows: 95°C for 30 s for activation, and then 40 cycles of 95°C for 10 s, 60°C for 30 s, 95°C for 15 s, 60°C for 60 s, and 95°C for 15 s. All experiments were performed in triplicate. GAPDH was set as a reference gene [21, 22]. Relative expression of the gene was calculated using the 2^−*ΔΔ*Ct^ method. The primers were designed using Primer 3 software, and the efficiency of all primer pairs was better than 95% [23]. The sequences of primers are listed in Table S2.

### 2.7. Analysis of the Predictive Value of Biomarkers

Receiver operating characteristic (ROC) analysis was used to evaluate the diagnostic values in the MedCalc application (version 19.1.3). The area under the ROC curve (AUC) value was considered the diagnostic index.

### 2.8. Statistical Analysis

All statistics were analysed using GraphPad Prism software (version 6.02). The data were presented as mean ± SD, and the Student's *t*-test was used to determine differences between the two groups. *P* < 0.05 was considered a significant difference.

## 3. Results

### 3.1. The Characteristics of the Study Population

In this study, a total of 8 blood samples from 8 volunteers (4 healthy controls, 4 HF patients) were randomly selected for RNA sequencing, and 100 samples (50 healthy controls, 50 HF patients) were enrolled as validation. The clinical information of both study groups is displayed in Table 1. The clinical information of all individuals involved in RNA sequencing is presented in Table S3 and Figure S1. There is no significant differences in physical characteristics between the two groups. The level of blood pressure, heart rate, haemoglobin, CK, HDL, apolipoprotein, creatinine, uric acid, glucose, and MYO showed no difference between the two groups. However, HF patients presented higher LDH, CHO, TG, LDL, and NEFA contents. Moreover, HF patients also had higher CKMB, hsTNT, and NT-proBNP levels and lower LVEF levels. Among the HF subjects, the number of NYHA grade II patients accounted for 20%, grade III for 58%, and grade IV for 22%.

### 3.2. The Profile of Differentially Expressed lncRNAs and mRNAs between HF and Control Groups

**Figure 2:**
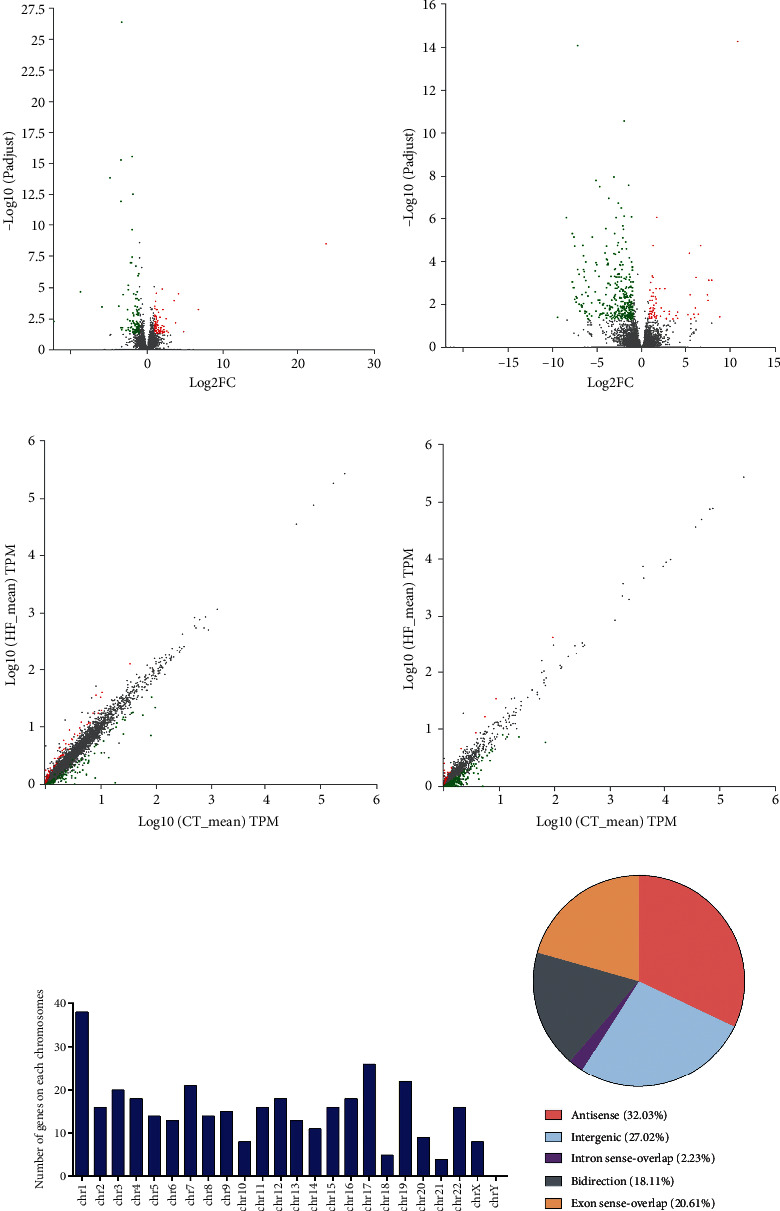
Sequencing profiles of differentially expressed (DE) lncRNAs and mRNAs in HF patients and healthy controls. Volcano plots show DE-mRNA (a) and DE-lncRNA (b) in the two groups. The *x*-axis shows the fold change (FC) in gene expression difference between the two samples, and the *y*-axis shows the *P* value. Scatter plot of DE-mRNAs (c) and DE-lncRNAs (d) expression profiles in the two groups. The *x*-axis shows the expression of the gene in the control sample, and the *y*-axis shows the expression of the gene in the HF sample. The red and green points represent up- and downregulated mRNA/lncRNAs, respectively. (e) Chromosomal distribution of DE-lncRNAs. (f) The classification of DE-lncRNAs.

**Figure 3:**
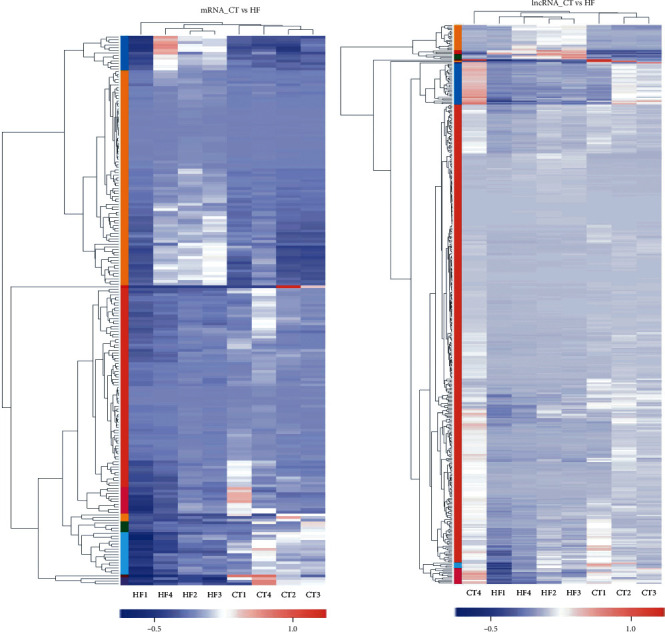
Gene set clustering of mRNA (a) and lncRNA (b) expression profiles in the two groups. Each column represents a sample, and each row represents a gene. The colour in the figure represents the size of the gene expression in the sample. Red represents the high expression of the gene in the sample, while blue represents the low expression. HF: heart failure; CT: healthy control.

In order to recognize the dysregulated lncRNAs and their potential pathological role in HF, RNA sequencing was performed and the lncRNA and mRNA expression profiles were analysed. We found a total of 207 mRNAs were differentially expressed between the two groups (Figures 2(a) and 2(c)). Among them, 93 were upregulated and 114 were downregulated. In addition, a total of 422 lncRNAs showed significantly different expression levels between the two groups, of which 80 were upregulated and 342 were downregulated. Volcano and scatter plots are shown in Figures 2(b) and 2(d). As shown in Figures 3(a) and 3(b), the hierarchical clustering analysis revealed the regulatory profiles of mRNA and lncRNAs between the CT and HF groups. The top-ten dysregulated mRNAs and lncRNAs are, respectively, shown in Tables 2 and 3.

The chromosome distribution of dysregulated lncRNAs is displayed in Figure 2(e). Chromosome 1 presented the greatest number of lncRNAs, followed by chromosome 17 and chromosome 19. According to the position of lncRNAs relative to protein-coding genes on the genome, lncRNAs can be divided into five types: exon-sense overlap, intergenic, antisense, intron sense overlap, and bidirectional. Antisense and intergenic lncRNAs accounted for a large proportion (Figure 2(f)).

### 3.3. Functional Enrichment Analysis

**Figure 4:**
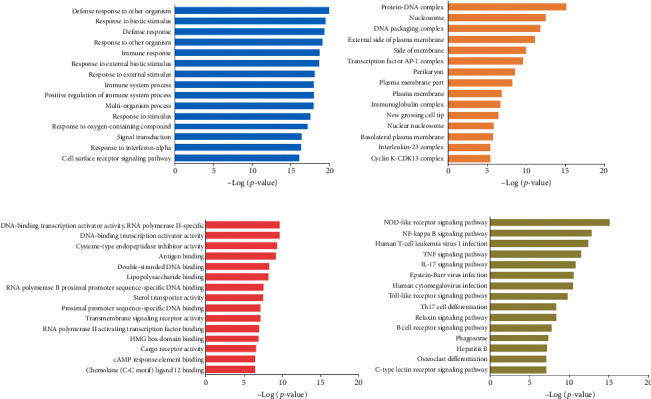
Functional enrichment analysis of DE-mRNAs in HF. The top 15 GO terms of biological process (a), cellular component (b), and molecular function (c). (d) The top 15 KEGG terms.

We performed GO and KEGG analyses to determine the coexpression of mRNAs. The top 15 significantly enriched GO terms are shown in Figure 4. The aberrantly expressed mRNA at BP levels was associated with defence responses to other organisms, response to biotic stimulus, immune response, response to external biotic stimulus, response to external stimulus, immune system process, response to oxygen-containing compound, signal transduction, response to interferon-alpha, and cell surface receptor signalling pathway (Figure 4(a)). In terms of CCs, the pathways were found to be related to the protein-DNA complex, DNA packaging complex, external side of plasma membrane, side of membrane, transcription factor AP-1 complex, plasma membrane part, plasma membrane, immunoglobulin complex, new growing cell tip, nuclear nucleosome, basolateral plasma membrane, interleukin-23 complex, and cyclin K-CDK13 complex (Figure 4(b)). In the category of MF, the mRNAs were enriched in the RNA polymerase II-specific, DNA-binding transcription activator activity, cysteine-type endopeptidase inhibitor activity, antigen binding, double-stranded DNA binding, lipopolysaccharide-binding, RNA polymerase II proximal promoter sequence-specific DNA binding, sterol transporter activity, proximal promoter sequence-specific DNA binding, transmembrane signalling receptor activity, RNA polymerase II activating transcription factor binding, HMG box domain binding, cargo receptor activity, cAMP response element binding, and chemokine (C-C motif) ligand 12 binding (Figure 4(c)).

Furthermore, KEGG analysis indicated that dysregulated mRNAs were associated with the NOD-like receptor signalling pathway, human T-cell leukaemia virus 1 infection, NF-*κ*B signalling pathway, TNF signalling pathway, IL-17 signalling pathway, Toll-like receptor signalling pathway, Th17 cell differentiation, relaxin signalling pathway, B cell receptor signalling pathway, phagosome, osteoclast differentiation, hepatitis B, and C-type lectin receptor signalling pathway (Figure 4(d)).

### 3.4. Construction of Coexpression and Target Prediction Network

**Figure 5:**
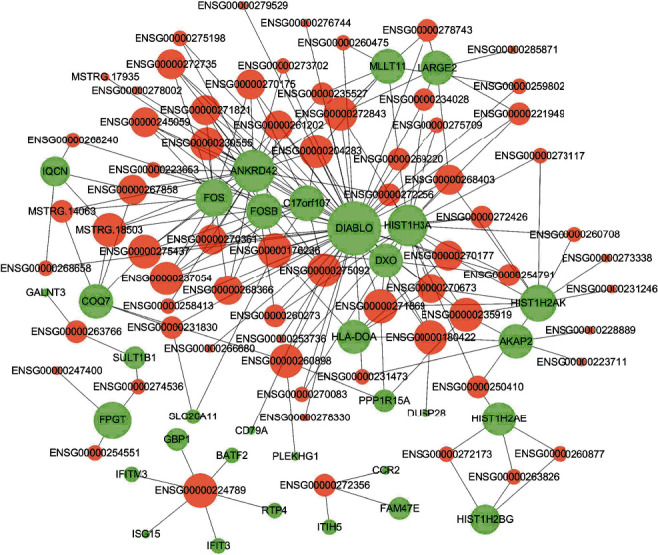
The interaction network of lncRNA-coexpressed mRNA pairs between HF and control. The orange circles represent lncRNAs, while the green circles represent coexpressed mRNA. The size of each circle represents the degree of centrality of the gene in the network, defined as the link numbers of the node.

To explore the interaction of aberrantly expressed genes in HF, a coexpression network was constructed based on correlation analysis. A total of 328 pairs of lncRNA-mRNA interactions (including 125 lncRNAs and 70 mRNAs) were selected, and the network was constructed with Cytoscape software (Figure 5). For example, lncRNA ENSG00000224789 was an upregulated novel transcript which could interact with GBP1, BATF2, IFITM3, RTP4, IFIT3, and ISG15. HIST1H2AK was associated with ENSG00000260708, ENSG00000273338, ENSG00000231246, ENSG00000235919, ENSG00000271869, ENSG00000273117, and ENSG00000272426. Other lncRNAs (such as ENSG00000204283, ENSG00000275092, and ENSG00000272843) and mRNAs (DIABLO, HIST1H3A, ANKRD42, C17ORF107, FOSB, FOS, and HLA-DOA) also demonstrated interactions. These data suggest that these genes may play critical roles in the coexpression network. All the interactions of lncRNA and mRNA are listed in Table S4.

**Figure 6:**
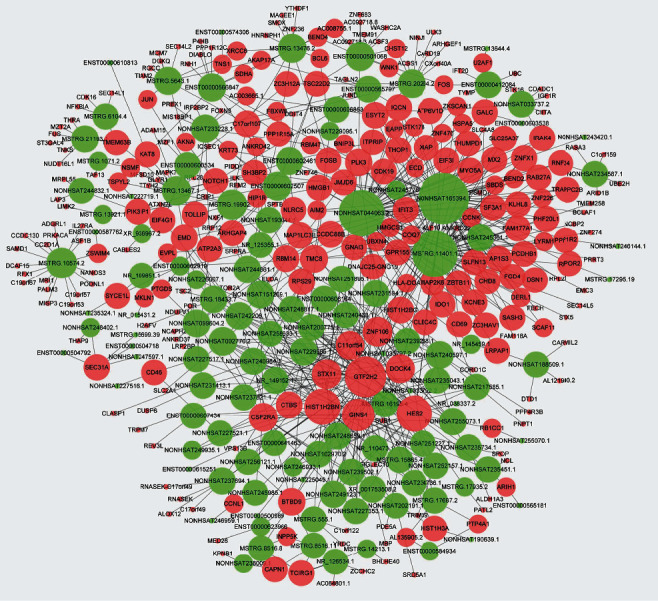
The network of differentially expressed lncRNAs and potential targets in HF. the green nodes represent lncRNAs, and the orange nodes represent mRNAs. The size of the circles represents the degree of centrality of the gene in the network, defined as the link numbers of the node.

We performed cisregulation and transregulation to analyse the potential targets of differentially expressed lncRNAs. As shown in Figure 6, several lncRNAs were found to be associated with multiple target genes, including HISTH2BN, GTF2H2, HES2, DOCK4, and STX11. These associations may provide important references for the further study of lncRNAs. The detailed prediction information is listed in Table S5.

### 3.5. Validation and Diagnostic Value Analysis of Key lncRNAs

**Figure 7:**
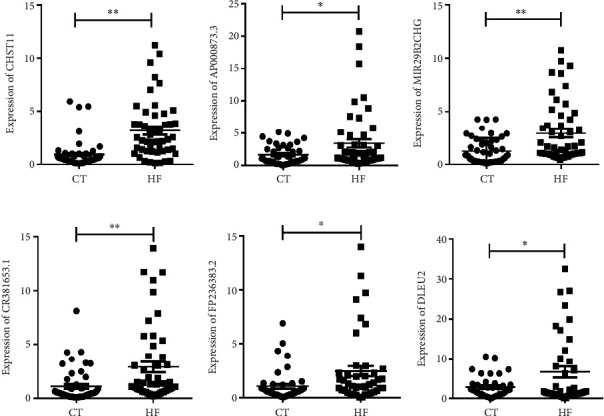
The validation of differentially expressed lncRNAs (a) CHST11, (b) AP000873.3, (c) MIR29B2CHG, (d) CR381653.1, (e) FP236383.2, and (f) DLEU2 by RT-qPCR. HF: heart failure; CT: healthy control. ^∗^*P* < 0.05, ^∗∗^*P* < 0.01.

Based on sequencing results, top-six upregulated lncRNAs (CHST11, MIR29B2CHG, AP000873.3, CR381653.1, FP236383.2, and DLEU2) were selected to detect expression in all the remaining samples enrolled in this study. As shown in Figure 7, the expression of those lncRNAs was indeed significantly increased in HF patients. These results were consistent with the sequencing data.

**Figure 8:**
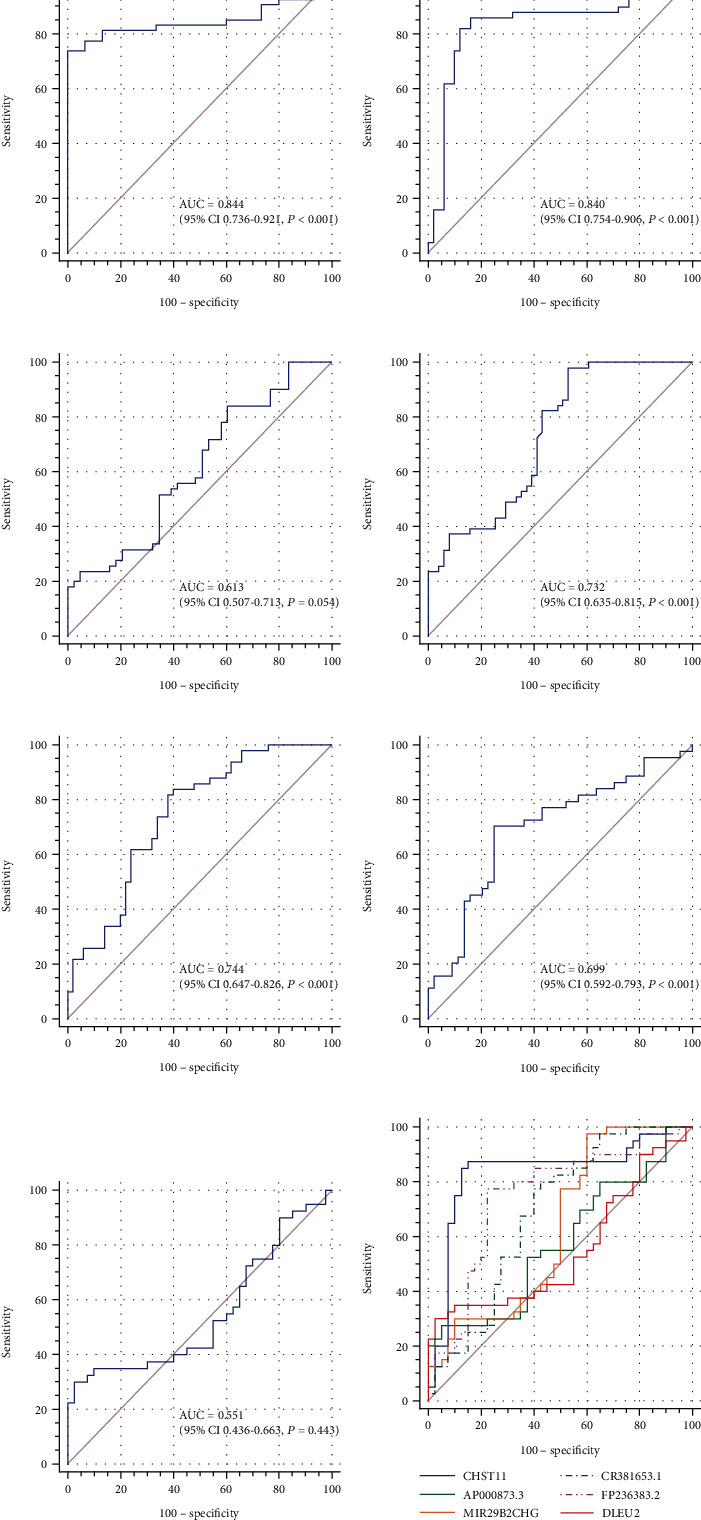
ROC curve analysis of (a) NT-proBNP, (b) CHST11, (c) AP000873.3, (d) MIR29B2CHG, (e) CR381653.1, (f) FP236383.2, (g) DLEU2, and (h) circulating lncRNAs. The AUC value and *P* value are listed in the box.

A ROC curve analysis was carried out to evaluate the diagnosis ability of HF (Figure 8, Table S6). The AUC values of NT-proBNP, CHST11, MIR29B2CHG, AP000873.3, CR381653.1, FP236383.2, and DLEU2 were 0.844, 0.84, 0.732, 0.613, 0.744, 0.699, and 0.551, respectively. However, the *P* value of AP000873.3 and DLEU2 was 0.054 and 0.443, which were greater than 0.05.

## 4. Discussion

lncRNAs account for the majority of ncRNAs, which were previously thought to be the by-product of RNA polymerase II transcription and have no biological functions. However, a large number of lncRNAs have been confirmed to regulate cellular processes and be related to the occurrence and development of many diseases [24–26]. HF is considered the common outcome of various myocardial diseases, such as cardiomyopathy, myocardial infarction, and hypertension [27]. Despite advances in pharmacology and medical technology, a large proportion of patients with heart disease progress to advanced HF [28, 29]. The prevalence of HF has reached 20% at the age of 40, and the incidence increases with age [30]. Previous studies have reported that many aberrantly expressed lncRNAs play a critical role in the pathogenesis and progression of cardiac hypertrophy and myocardial injury [31–33]. Yang et al. reported that the lncRNA MIAT could affect the handling of calcium and contractile function, resulting in cardiac myocyte remodelling and hypertrophy [11].

Blood is an easily accessible tissue and is associated with the occurrence of CVDs and the expression of their risk factors [34]. It can also provide information about a patient's status and can be expanded to very large sample sizes for screening biomarkers [21, 35, 36]. In this study, we collected 100 blood samples from 100 volunteers, including 50 HF patients and 50 healthy controls. A total of 207 mRNAs and 422 lncRNAs were differentially expressed in the HF group compared with the controls. Many new differential genes were found in our study, and these genes have not been comprehensively verified or studied; hence, our study provides a comprehensive differential gene expression map that may help understand part of the regulatory mechanism of HF.

In order to understand the function of differentially expressed lncRNAs in HF, we performed GO and KEGG analyses on its related mRNAs. And then, the functional enrichment analysis showed that the differentially expressed genes were mainly involved in immune response, DNA packaging complex, DNA-binding transcription activator activity, RNA polymerase II-specific, and related to the biological processes of the plasma membrane and cell membrane. These pathways seemed to be associated with myocardial hypertrophy, cardiac aging, and HF. HF is the end-stage of heart disease, and its pathological factors involve cardiac hypertrophy, apoptosis, functional impairment, myocardial fibrosis, and cardiac remodelling [37–40]. Similar pathways were analysed by KEGG pathway analysis. The top 15 enrichment pathways included the NOD-like receptor signalling pathway, human T-cell leukaemia virus 1 infection, NF-kappa B signalling pathway, TNF signalling pathway, and IL-17 signalling pathway. These data demonstrated that the dysregulated genes were involved in immune and inflammatory responses. Our results were consistent with previous studies, which reported that inflammatory responses and apoptosis were associated with the pathogenesis of HF [41–43].

lncRNAs usually act as sponges of the other genes, including miRNAs and mRNAs, to exert their regulatory functions. Based on the results of RNA-sequencing, we performed correlation analysis of lncRNAs and mRNAs and constructed the coexpression network with bioinformatic tools. Our results found a connection between several mRNAs and the dysregulated lncRNAs. To cite one, we found DIABLO possessing a high degree of connection. Additionally, the expression of DIABLO was associated with cardiomyocyte apoptosis, which is induced by cardiac ischemia [44]. Posttranslational protein modification plays an emerging and vital role in the occurrence and development of cardiovascular diseases [45]. HIST1H3A has been reported to regulate protein acetylation in cardiovascular pathologies [46]. Moreover, ischemia-reperfusion injury could affect the localization of FOSB in the cardiac myocyte [47]. In the cisregulation and transregulation networks, we found several mRNAs that could be potential targets of lncRNAs. The high degree of centrality of the mRNA included FOSB, DOX4, and CDK19. Among those genes, CDK19 was a component of the transcriptional mediator kinase module regulating gene transcription [48]. Recent studies also found dysregulated CDK19 to be associated with coronary artery diseases [49]. EGR1 is a key gene in the process of cell differentiation and mitogenesis. Upregulated EGR1 can trigger inflammatory responses and cell death [50]. The high expression of EGR1 was found in the HF group, and this result was consistent with a previous study [35]. Although we found that several lncRNAs were related to specific mRNAs, further investigations are still needed to verify their relationship.

We selected the top six upregulated lncRNAs (CHST11, MIR29B2CHG, AP000873.3, CR381653.1, FP236383.2, and DLEU2) and detected their expression in all samples. The validation results were consistent with those of the sequencing data. lncRNA CHST11 is a transcript variant of carbohydrate sulfotransferase 11, and the results of sequencing and RT-qPCR were both upregulated in the HF group. Previous studies reported that MIR29B2CHG dysregulation is associated with colorectal cancer, pulmonary adenocarcinoma, breast cancer, and adrenocortical carcinoma [51–54]. Interestingly, in this study, we found the expression levels of MIR29B2CHG to be upregulated in the HF group. DLEU2 has been reported to play a crucial role in the progression of cancers such as the case of liver cancer and endometrial cancer [55, 56]. Some researchers have also reported that DLEU2 could regulate the differentiation of the skeletal muscle [57]. The lncRNAs CR381653.1, AP000873.3, and FP236383.2 are novel upregulated transcripts being reported for the first time in the present study. lncRNA AP000873.3 is an exon-sense overlap lncRNA and is located on chromosome 11. On the other hand, lncRNAs CR381653.1 and FP236383.2 are both intergenic lncRNAs located on chromosome 21.

We found the expression of these lncRNAs to be highly different between the HF and control groups. According to previous studies, the highly dysregulated lncRNAs in cardiovascular diseases could be considered biomarker of these diseases [42, 58]. We hypothesized that the expression of these lncRNAs was related to the pathogenesis of HF. So, we further performed ROC curve analyses to assess their diagnostic value. Moreover, we also assessed the diagnostic value of NT-proBNP. The results showed that the AUC value of lncRNAs CHST11, CR381653.1, MIR29B2CHG, and FP236383.2 were all above than 0.6, with the exception of CHST11 whose value was close to that of NT-proBNP. Conversely, the AUC values of AP000873.3 and DLEU2 were 0.613 and 0.551, respectively, and their *P* values were greater than 0.5. Although the expression differences between the two lncRNAs were significant, there was no significant potential relationship between their expression levels and HF in terms of diagnostic value, which we believe is due to the insufficient sample size. NT-proBNP levels are known to be affected by many factors, such as age, drug intervention, obesity, tachycardia, renal insufficiency, sepsis, chronic obstructive pulmonary disease, and cirrhosis [59, 60]. Altogether, these results suggest that these lncRNAs may have a potential value as a diagnostic indicator for HF.

In an earlier study, Liu et al. found that RNA sequencing technology had better resolution and lower error rates compared to other analysis methods while requiring a smaller sample size [61]. Zheng et al. reported lncRNA GAS5 to be downregulated in HF based on bioinformatic analysis. In addition, GAS5 may functions as a competing endogenous RNA through modulating miRNAs and mRNAs in the progression of HF [62]. Chen et al. revealed the coexpression network of mRNAs and lncRNAs in pressure overload-induced HF by sequencing rat models [42]. At present, we selected 8 samples (4 HF and 4 controls) for sequencing analysis, while the differently expressed lncRNAs were detected and identified in 100 samples. We found that selected lncRNAs have high discrimination between HF and controls. These results suggest that these novel lncRNAs may function as potential biomarkers for HF.

However, this study also had several limitations. Even though the sample size was large compared to many previous studies, these lncRNAs still need to be further analysed in even larger-scale studies. All the participants in this study were Chinese, and thus, these findings should be confirmed in other populations. During the sample inclusion process, we included HF patients based on the combined clinical and lab test diagnosis, without being able to exclude the heterogeneity in their etiology, NYHA (New York Heart Association Class) class and/or HF type. In this current study, NYHA grade III and HFmrEF (HF with mildly reduced EF) types account for the majority of the HF participants. In addition, the underlying molecular mechanisms of how these dysregulated lncRNAs regulate HF still need to be explored.

## 5. Conclusions

In conclusion, our work revealed the expression profiles of differentially expressed lncRNAs and mRNAs between the HF and control groups by transcriptome sequencing. Several differentially expressed mRNAs are reported to be involve in various biological pathways related to the pathogenesis of HF. The lncRNA-mRNA coexpression network was conducted to find the hub genes related to the pathology of HF. Moreover, combined sequencing and validation analyses report several lncRNAs with high expression levels that present a confident diagnostic value for HF. These findings provide new insights for understanding the underlying mechanisms and therapeutic targets of HF.

## Figures and Tables

**Table 1:** Clinical characteristics of the healthy control and HF patients.

	CT	HF	*P* value
Male/female (*n*/*n*)	18/32	21/29	0.1695
Age (year)	68.52 ± 0.89	70.84 ± 1.17	0.1197
BMI (kg/m2)	22.94 ± 3.29	24.95 ± 2.36	0.9913
Heart rate (time/minute)	73.80 ± 1.42	71.14 ± 1.45	0.1931
SBP (mmHg)	126.8 ± 1.18	130.0 ± 1.29	0.1617
DBP (mmHg)	73.83 ± 1.48	75.49 ± 1.29	0.3995
Hemoglobin (g/L)	129.1 ± 3.58	142.1 ± 5.24	0.0633
LDH (U/L)	144.3 ± 5.30	162.2 ± 4.97	0.0173
CK (U/L)	73.56 ± 5.54	75.48 ± 7.36	0.8359
CHO (mmol/L)	2.94 ± 0.42	4.98 ± 0.43	0.0072
TG (mmol/L)	1.19 ± 0.17	2.21 ± 0.26	0.0099
LDL (mmol/L)	2.25 ± 0.09	3.32 ± 0.35	0.0337
HDL (mmol/L)	1.54 ± 0.22	1.34 ± 0.17	0.515
ApoA (g/L)	1.38 ± 0.04	1.42 ± 0.05	0.4883
ApoB (g/L)	1.12 ± 0.06	1.04 ± 0.04	0.3306
NEFA (mmol/L)	0.48 ± 0.03	0.74 ± 0.05	0.0026
Creatinine (*μ*mol/L)	70.85 ± 2.29	80.05 ± 4.78	0.0959
Uric acid (*μ*mol/L)	316.40 ± 16.90	299.20 ± 14.21	0.4389
Glucose (mmol/L)	5.32 ± 0.22	6.12 ± 0.42	0.1354
CKMB (ng/ml)	1.08 ± 0.10	4.42 ± 0.88	0.0442
MYO (ng/ml)	39.42 ± 3.51	57.97 ± 10.73	0.516
HsTNT (ng/L)	8.93 ± 0.77	12.31 ± 0.84	0.0183
NT-proBNP (ng/L)	62.83 ± 5.82	711.20 ± 32.30	<0.0001
Echocardiographic parameters			
LVEF (%)	62.9 ± 2.96	48.7 ± 1.44	<0.0001
LVFS (%)	33.97 ± 1.80	22.50 ± 0.92	<0.0001
LVDd (mm)	46.30 ± 0.93	58.85 ± 1.32	<0.0001
LVDs (mm)	30.93 ± 0.43	39.32 ± 1.31	<0.0001
IVST (mm)	8.50 ± 0.21	11.38 ± 1.28	<0.0001
LVPWd (mm)	7.83 ± 1.20	9.12 ± 0.25	<0.0001
ESV (ml)	55.17 ± 0.93	76.21 ± 2.60	<0.0001
EDV (ml)	112.0 ± 1.81	139.1 ± 3.01	<0.0001
NYHA class			
II (*n*, %)	0	10, 20%	
III (*n*, %)	0	29, 58%	
IV (*n*, %)	0	11, 22%	
HF type			
HFrEF (*n*, %)	0	12, 24%	
HFmrEF (*n*, %)	0	29, 58%	
HFpEF (*n*, %)	0	9, 18%	
Etiology			
Hypertension (*n*, %)	0	14, 28%	
Myocardial infarction (*n*, %)	0	11, 22%	
Diabetes mellitus (*n*, %)	0	13, 26%	
Coronary artery disease (*n*, %)	0	9, 18%	
Valvular heart disease (*n*, %)	0	3, 6%	

BMI: body mass index; SBP: systolic blood pressure; DBP: diastolic blood pressure; LDH: lactate dehydrogenase; CK: creatine kinase; CHO: cholesterol; TG: total triglyceride; LDL: low-density lipoprotein; HDL: high-density lipoprotein; ApoA: apolipoprotein A; ApoB: apolipoprotein B; NEFA: nonestesterified fatty acid; CKMB: creatine kinase MB; MYO: myoglobin; hsTNT: high sensitivity troponin T; NT-proBNP: N-terminal pro-B type natriuretic peptide; LVEF: left ventricular ejection fraction; LVFS: left ventricular fractional shortening; LVDd: left ventricular diameter at end-diastole. LVDs: left ventricular diameter at end-systole; IVST: interventricular septum thickness; LVPWd: left ventricular posterior wall diameter; ESV: end-systolic volume; EDV: end-diastolic volume; NYHA: New York Heart Association Class; HFrEF: HF with reduced EF; HFmrEF: HF with mildly reduced EF; HFpEF: HF with preserved EF.

**Table 2:** Top 10 dysregulated differentially expressed mRNAs.

mRNA ID	mRNA name	Log2FC	*P* value	Regulation
ENSG00000260287	TBC1D3G	4.874796692	0.001087542	Up
ENSG00000172967	XKR3	4.179624297	7.09E-08	Up
ENSG00000239704	CDRT4	3.564681164	3.30E-07	Up
ENSG00000159189	C1QC	2.788040027	0.001359143	Up
ENSG00000182111	ZNF716	2.571515863	0.000959598	Up
ENSG00000115457	IGFBP2	2.49533699	2.50E-05	Up
ENSG00000104728	ARHGEF10	2.28083052	0.001103817	Up
ENSG00000110203	FOLR3	2.265093426	0.000606649	Up
ENSG00000078081	LAMP3	2.223730623	0.001339717	Up
ENSG00000119917	IFIT3	2.214722509	0.000249811	Up
ENSG00000176020	AMIGO3	-5.868097367	1.37426E-06	Down
ENSG00000125740	FOSB	-4.857959145	4.52716E-18	Down
ENSG00000125968	ID1	-3.422339219	0.000307407	Down
ENSG00000153234	NR4A2	-3.358003116	4.83417E-16	Down
ENSG00000177606	JUN	-3.270105417	3.33777E-31	Down
ENSG00000156966	B3GNT7	-3.200020621	0.000369482	Down
ENSG00000205710	C17orf107	-3.062020349	8.359E-08	Down
ENSG00000123689	G0S2	-2.508055643	3.42221E-06	Down
ENSG00000100003	SEC14L2	-2.474567404	3.4328E-05	Down
ENSG00000080573	COL5A3	-2.45784054	0.000566826	Down

FC: Fold change.

**Table 3:** Top 10 dysregulated differentially expressed lncRNAs.

lncRNA ID	lncRNA name	Log2FC	*P* value	Regulation
NONHSAT030431.2	CHST11	10.85008805	4.42E-19	Up
NR_125355.1	AP000873.3	6.726598259	4.18E-08	Up
NONHSAT227521.1	MIR29B2CHG	5.500291789	1.24E-07	Up
XR_001754947.1	CR381653.1	2.769049361	1.73E-05	Up
NR_152571.1	DLEU2	2.683433828	0.000490854	Up
NONHSAT190639.1	FP236383.2	2.131295637	1.71E-05	Up
ENST00000641463	AL596257.1	1.876536112	0.000997036	Up
NONHSAT242206.1	DGUOK-AS1	1.837906331	0.000990179	Up
MSTRG.13467.1	FP671120.2	1.814340444	1.07E-09	Up
NONHSAT253292.1	TRG-AS1	1.797946656	0.000131498	Up
NR_104487.1	ZDHHC20-IT1	-9.439932916	0.001417839	Down
NONHSAT173188.1	CBFB	-8.456016875	1.31E-09	Down
ENST00000594927	AC123912.2	-7.831658257	9.1767E-09	Down
ENST00000608450	AC004854.2	-7.785597503	7.43E-06	Down
NONHSAT161885.1	ATF7IP	-7.66602433	1.59255E-05	Down
NONHSAT235451.1	TMEM202-AS1	-7.660442444	1.46E-08	Down
NONHSAT225182.1	AL078459.1	-7.58664362	1.50902E-05	Down
NONHSAT033737.2	CDADC1	-7.549563565	5.36E-08	Down
MSTRG.10794.2	ZNF257	-7.539906253	8.61862E-05	Down
MSTRG.10792.1	AC123912.4	-7.374615363	1.21E-04	Down

FC: fold change.

## Data Availability

The data used to support the findings of this study have been deposited in the NCBI's Sequence Read Archive (SRA), reference number (BioProject ID: PRJNA859795).

## Supplementary Materials

Supplementary Materials Table S1 shows the detailed sequencing data of the samples. The primers are shown in Table S2. Table S3 shows the clinical information for all individuals involved in RNA sequencing. Table S4 and Table S5 show the coexpression network data and the data of predicted target genes of lncRNAs, respectively. Table S6 shows the ROC curve analysis of NT-proBNP and lncRNAs. Figure S1 shows the echocardiograms of all individuals involved in RNA sequencing.
