# Supplementary material for: Evaluation of lncRNA Expression Pattern and Potential Role in Heart Failure Pathology
Source: Dis Markers. 2023 Jul 12;2023:2369352. doi: 10.1155/2023/2369352 (PMC10356452; doi:10.1155/2023/2369352)
Supplement: Supplementary Materials — Table S1 shows the detailed sequencing data of the samples. The primers are shown in Table S2. Table S3 shows the clinical information for all individuals involved in RNA sequencing. Table S4 and Table S5 show the coexpression network data and the data of predicted target genes of lncRNAs, respectively. Table S6 shows the ROC curve analysis of NT-proBNP and lncRNAs. Figure S1 shows the echocardiograms of all individuals involved in RNA sequencing. [file 2369352.f1.docx]

**Supplementary Materials**

Table S1. The detailed sequencing data of the samples

| Sample | Raw reads | Raw bases | Error rate (%) | Q20(%) | Q30(%) | GC content (%) |
| --- | --- | --- | --- | --- | --- | --- |
| CT1 | 147138714 | 22217945814 | 0.015 | 97.85 | 94.66 | 53.64 |
| CT2 | 142763210 | 21557244710 | 0.017 | 97.95 | 94.83 | 55.31 |
| CT3 | 155045060 | 23411804060 | 0.016 | 97.77 | 94.5 | 55.65 |
| CT4 | 145531412 | 21975243212 | 0.017 | 97.72 | 94.42 | 53.84 |
| HF1 | 140256760 | 21178770760 | 0.017 | 97.89 | 94.76 | 57.23 |
| HF2 | 153801488 | 23224024688 | 0.016 | 97.86 | 94.72 | 53.53 |
| HF3 | 152912696 | 23089817096 | 0.016 | 97.78 | 94.64 | 52.79 |
| HF4 | 150568250 | 22735805750 | 0.016 | 97.87 | 94.8 | 54.59 |

Table S2. The primer of long non-coding RNA and GAPDH in the validation

| Gene ID | Gene | Primer | Sequences | Product length (bp) | Ta (℃) |
| --- | --- | --- | --- | --- | --- |
| ENSG00000171310 | CHST11 | Forward | TGGGGGCATGAATGTTCTCC | 184 | 55.4 |
|  |  | Reverse | TGCAAGGGACTTTCAGGTCG |  |  |
| ENSG00000203709 | MIR29B2CHG | Forward | TACTGCCGGATAGAGTGGCT | 180 | 55.4 |
|  |  | Reverse | TTCCAGTAAGCAGCCTGGTG |  |  |
| ENSG00000231607 | DLEU2 | Forward | CGCCATTTTCGAGTGATGCC | 181 | 55.4 |
|  |  | Reverse | ACAGGTCAAAACCGACTGCG |  |  |
| ENSG00000254551 | AP000873.3 | Forward | TGACTTGAGAAGGGTTCCAGG | 139 | 55.5 |
|  |  | Reverse | GTGCTGCCCAATGTAAAGGG |  |  |
| ENSG00000278932 | CR381653.1 | Forward | CGCCATTTTCCTCCGGAAGTG | 159 | 54.5 |
|  |  | Reverse | TCCCACAACTAGGGAACACTG |  |  |
| ENSG00000280614 | FP236383.2 | Forward | CGGCGTCCCCCAACTTCTTA | 191 | 55.5 |
|  |  | Reverse | GCAATCCCCGATCCCCATCA |  |  |
| 2597 | GAPDH | Forward | CTGACTTCAACAGCGACACC | 217 | 55.6 |
|  |  | Reverse | TGCTGTAGCCAAATTCGTTGT |  |  |

Ta, annealing temperature.

Table S3 Clinical information for all individuals involved in RNA sequencing.

| Group | Gender | Age  (year) | Height  (cm) | Weight  (kg) | BMI  (kg/m2) | Smoking history | Drinking history | Family history of CVD | Heart rate  (Time/minute) | SBP  (mmHg) | DBP  (mmHg) | Hemoglobin  (g/L) | LDH  (U/L) |
| --- | --- | --- | --- | --- | --- | --- | --- | --- | --- | --- | --- | --- | --- |
| CT1 | male | 72 | 175 | 75 | 24.49 | no | no | no | 83 | 120 | 70 | 138 | 142 |
| CT2 | male | 73 | 173 | 63 | 21.05 | no | no | no | 77 | 126 | 74 | 147 | 137 |
| CT3 | female | 74 | 168 | 65 | 23.03 | no | no | no | 72 | 134 | 77 | 119 | 125 |
| CT4 | female | 73 | 164 | 54 | 20.08 | no | no | no | 83 | 130 | 71 | 122 | 155 |
| HF1 | male | 72 | 173 | 70 | 23.39 | no | no | no | 80 | 126 | 80 | 139 | 141 |
| HF2 | male | 73 | 170 | 72 | 24.91 | no | no | no | 75 | 124 | 75 | 107 | 156 |
| HF3 | female | 74 | 160 | 60 | 23.44 | no | no | no | 76 | 117 | 73 | 118 | 155 |
| HF4 | female | 73 | 169 | 65 | 23.46 | no | no | no | 88 | 134 | 70 | 117 | 146 |

| Group | CK  (U/L) | CHO (mmol/L) | TG (mmol/L) | LDL (mmol/L) | HDL (mmol/L) | ApoA  (g/L) | ApoB  (g/L) | NEFA  (mmol/L) | Creatinine  (umol/L) | Uric acid  (umol/L) | Glucose  (mmol/L) | CKMB  (ng/ml) | MYO  (ng/ml) |
| --- | --- | --- | --- | --- | --- | --- | --- | --- | --- | --- | --- | --- | --- |
| CT1 | 61 | 4.77 | 1.12 | 1.32 | 1.08 | 1.50 | 1.03 | 0.32 | 71 | 281 | 4.61 | 1.56 | 23.00 |
| CT2 | 71 | 4.95 | 1.39 | 2.15 | 1.42 | 1.19 | 0.92 | 0.41 | 64 | 329 | 4.96 | 1.54 | 24.17 |
| CT3 | 76 | 4.20 | 1.41 | 2.06 | 0.85 | 1.48 | 1.06 | 0.25 | 86 | 263 | 5.35 | 1.97 | 33.42 |
| CT4 | 66 | 3.44 | 1.77 | 1.59 | 1.37 | 1.48 | 0.80 | 0.30 | 60 | 242 | 4.52 | 0.98 | 21.00 |
| HF1 | 85 | 4.60 | 2.86 | 3.28 | 1.42 | 1.50 | 0.92 | 0.41 | 64 | 295 | 6.24 | 2.95 | 34.06 |
| HF2 | 70 | 6.19 | 4.27 | 3.69 | 1.08 | 1.82 | 1.06 | 0.43 | 63 | 257 | 4.30 | 1.57 | 35.28 |
| HF3 | 66 | 4.95 | 5.44 | 1.99 | 0.83 | 1.48 | 0.97 | 0.57 | 50 | 182 | 13.33 | 2.15 | 30.27 |
| HF4 | 81 | 5.27 | 1.83 | 2.59 | 1.24 | 1.27 | 0.72 | 0.38 | 120 | 328 | 4.36 | 3.68 | 84.90 |

| Group | hsTNT  (ng/L) | NT-ProBNP  (ng/L) | LVEF  (%) | LVFS  (%) | LVDd  (mm) | LVDs  (mm) | IVST  (mm) | LVPWd  (mm) | ESV  (ml) | EDV  (ml) | NYHA class | HF type | etiology |
| --- | --- | --- | --- | --- | --- | --- | --- | --- | --- | --- | --- | --- | --- |
| CT1 | 9.05 | 112.1 | 68.6 | 38.2 | 44.0 | 27.2 | 8 | 8 | 60 | 87 | no | no | no |
| CT2 | 7.3 | 126.8 | 65.0 | 35.0 | 45.0 | 29.0 | 9 | 9 | 59 | 92 | no | no | no |
| CT3 | 7.63 | 118.8 | 61.1 | 32.2 | 38.8 | 26.3 | 8 | 8 | 39 | 65 | no | no | no |
| CT4 | 6.64 | 97.27 | 61.8 | 33.0 | 43.3 | 29.0 | 8 | 8 | 52 | 84 | no | no | no |
| HF1 | 10.96 | 1160 | 33.2 | 16.5 | 78.6 | 65.6 | 9 | 5 | 110 | 331 | IV | HFrEF | MI, VHD |
| HF2 | 9.86 | 516.9 | 42.0 | 20.6 | 49.4 | 39.2 | 11 | 10 | 48 | 115 | III | HFmrEF | CAD |
| HF3 | 12.89 | 451.6 | 44.3 | 22.1 | 51.2 | 29.9 | 8 | 8 | 44 | 116 | III | HFmrEF | MI, VHD |
| HF4 | 23.88 | 544.8 | 33.5 | 16.5 | 69.9 | 58.4 | 9 | 8 | 169 | 255 | IV | HFrEF | MI, VHD |

BMI, body mass index. CVD, cardiovascular diseases. SBP, systolic blood pressure. DBP, diastolic blood pressure. LDH: lactate dehydrogenase. CK: creatine kinase. CHO, cholesterol; TG, total triglyceride. LDL, low-density lipoprotein. HDL, high-density lipoprotein. ApoA: apolipoprotein A. ApoB: apolipoprotein B. NEFA: nonestesterified fatty acid. CKMB, creatine kinase MB. MYO, myoglobin. hsTNT, high sensitivity troponin T. NT-proBNP, N-terminal pro-B type natriuretic peptide. LVEF: left ventricular ejection fraction. LVEF, left ventricular ejection fraction. LVFS, left ventricular fractional shortening. LVDd, left ventricular diameter at end-diastole. LVDs, left ventricular diameter at end-systole. IVST, interventricular septum thickness. LVPWd, left ventricular posterior wall diameter. ESV, end-systolic volume. EDV, end-diastolic volume. NYHA, New York Heart Association Class. HFrEF, HF with reduced EF. HFmrEF, HF with mildly reduced EF. MI, myocardial infarction. CAD, coronary artery disease. VHD, valvular heart disease.


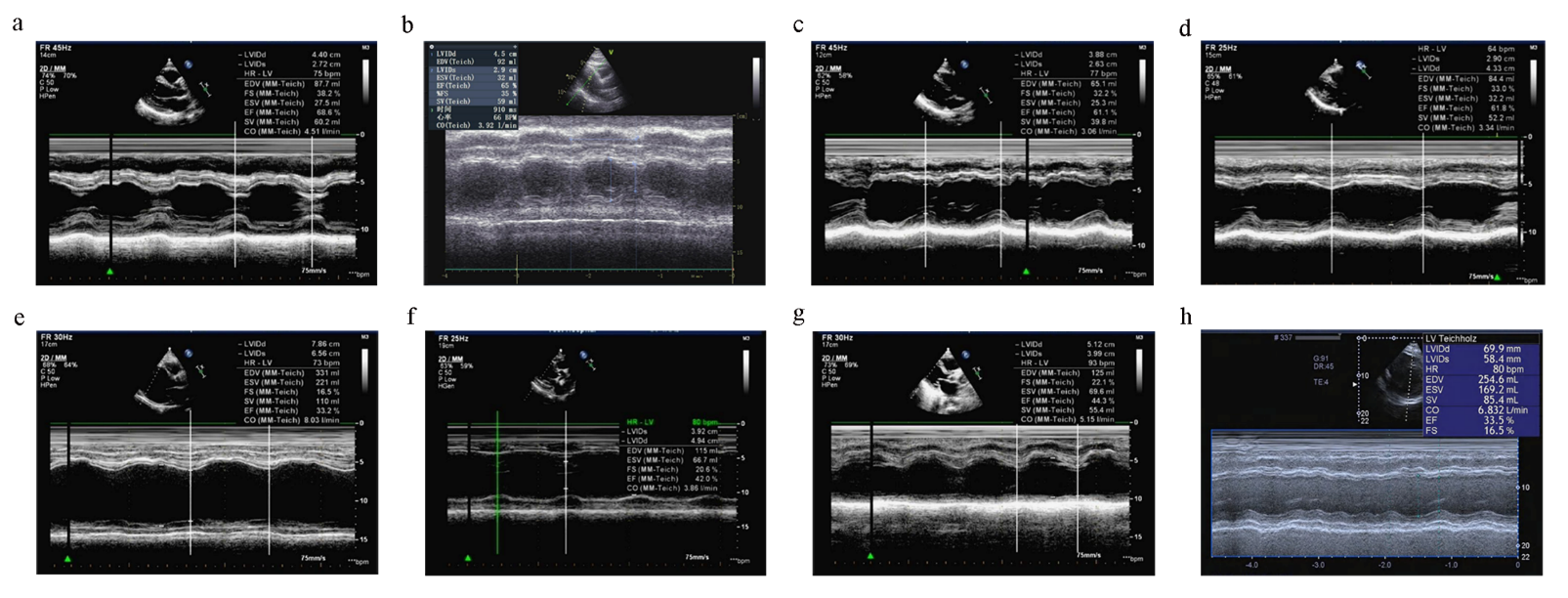


Figure S1. Echocardiograms of all individuals involved in RNA sequencing. (a) CT1, (b) CT2, (c) CT3, (d) CT4, (e) HF1, (f) HF2, (f) HF3, (h) HF4. CT, healthy control. HF, heart failure.

Table S4. Correlation analysis results between lncRNA and mRNA.

| lncRNA | mRNA | Cor | *p*-value |
| --- | --- | --- | --- |
| ENSG00000272843 | DIABLO | 0.996539 | 3.60E-05 |
| ENSG00000234028 | DIABLO | 0.99406 | 0.000142 |
| ENSG00000278743 | DIABLO | 0.991929 | 0.000293 |
| ENSG00000235527 | DIABLO | 0.99072 | 0.000388 |
| ENSG00000275198 | DIABLO | 0.986779 | 0.000735 |
| ENSG00000269220 | DIABLO | 0.986714 | 0.000741 |
| ENSG00000272256 | DIABLO | 0.985916 | 0.000809 |
| ENSG00000230555 | DIABLO | 0.984964 | 0.000912 |
| ENSG00000260273 | DIABLO | 0.984895 | 0.000915 |
| ENSG00000270177 | HIST1H3A | 0.984458 | 0.000966 |
| ENSG00000268403 | DIABLO | 0.984361 | 0.00098 |
| ENSG00000268366 | DIABLO | 0.983847 | 0.001028 |
| ENSG00000223653 | DIABLO | 0.983786 | 0.001033 |
| ENSG00000273702 | DIABLO | 0.981041 | 0.001367 |
| ENSG00000242828 | FPGT | 0.980482 | 0.001446 |
| ENSG00000237054 | DIABLO | 0.980243 | 0.001477 |
| ENSG00000259802 | DIABLO | 0.980211 | 0.001481 |
| ENSG00000272173 | HIST1H2AE | 0.979773 | 0.001544 |
| ENSG00000258413 | DIABLO | 0.979629 | 0.001562 |
| ENSG00000245059 | DIABLO | 0.979453 | 0.001584 |
| ENSG00000260101 | DIABLO | 0.979375 | 0.001592 |
| ENSG00000270175 | DIABLO | 0.979374 | 0.001592 |
| ENSG00000260475 | DIABLO | 0.979311 | 0.001598 |
| ENSG00000268240 | DIABLO | 0.979155 | 0.00162 |
| ENSG00000176236 | C17orf107 | 0.978655 | 0.001676 |
| ENSG00000226828 | DIABLO | 0.978307 | 0.001714 |
| ENSG00000221949 | DIABLO | 0.978032 | 0.001748 |
| ENSG00000275092 | DIABLO | 0.977487 | 0.001824 |
| ENSG00000267858 | DIABLO | 0.976918 | 0.001887 |
| ENSG00000271821 | DIABLO | 0.976803 | 0.001903 |
| ENSG00000223711 | AKAP2 | 0.976582 | 0.001933 |
| ENSG00000179840 | ZNF716 | 0.975599 | 0.002077 |
| ENSG00000270361 | DIABLO | 0.975312 | 0.002119 |
| ENSG00000179840 | FPGT | 0.975162 | 0.00214 |
| ENSG00000261202 | DIABLO | 0.974755 | 0.002197 |
| ENSG00000260898 | DIABLO | 0.974392 | 0.002248 |
| ENSG00000272173 | HIST1H2BG | 0.974105 | 0.002287 |
| ENSG00000268403 | HIST1H3A | 0.974053 | 0.002296 |
| ENSG00000275709 | DIABLO | 0.97393 | 0.002311 |
| ENSG00000253736 | DIABLO | 0.973278 | 0.002417 |
| ENSG00000271780 | DIABLO | 0.972565 | 0.002532 |
| ENSG00000235919 | AKAP2 | 0.972393 | 0.002553 |
| ENSG00000275437 | DIABLO | 0.972407 | 0.002553 |
| ENSG00000204283 | DIABLO | 0.97135 | 0.002699 |
| ENSG00000272735 | DIABLO | 0.971011 | 0.002755 |
| ENSG00000248544 | FPGT | 0.969074 | 0.003087 |
| ENSG00000270177 | DIABLO | 0.968012 | 0.003242 |
| ENSG00000203327 | CKLF-CMTM1 | 0.966609 | 0.003468 |
| ENSG00000255062 | HIST1H2AK | 0.965959 | 0.003592 |
| ENSG00000180422 | HLA-DOA | 0.965314 | 0.003708 |
| ENSG00000228786 | FPGT | 0.964927 | 0.0038 |
| ENSG00000234553 | MRC1 | 0.963197 | 0.004126 |
| ENSG00000203327 | MGAM2 | 0.963075 | 0.004145 |
| ENSG00000225855 | PPIAL4G | 0.962898 | 0.004187 |
| ENSG00000180422 | DXO | 0.962401 | 0.004262 |
| ENSG00000268658 | DIABLO | 0.962245 | 0.004297 |
| ENSG00000237803 | CKLF-CMTM1 | 0.961754 | 0.004384 |
| ENSG00000179840 | MGAM2 | 0.961178 | 0.004497 |
| ENSG00000270673 | HIST1H3A | 0.960191 | 0.004707 |
| ENSG00000176236 | SLC26A11 | 0.960107 | 0.004708 |
| ENSG00000179840 | C9orf66 | 0.959972 | 0.004731 |
| ENSG00000254551 | FPGT | 0.95909 | 0.004915 |
| ENSG00000254791 | DIABLO | 0.958856 | 0.004962 |
| ENSG00000233143 | MRC1 | 0.958082 | 0.00512 |
| ENSG00000247400 | FPGT | 0.956228 | 0.00549 |
| ENSG00000176236 | DIABLO | 0.956076 | 0.005515 |
| ENSG00000204283 | C17orf107 | 0.955741 | 0.005584 |
| ENSG00000250309 | MRC1 | 0.955226 | 0.005687 |
| ENSG00000231830 | DIABLO | 0.95485 | 0.005782 |
| ENSG00000233030 | FPGT | 0.954436 | 0.005865 |
| ENSG00000225979 | FPGT | 0.954362 | 0.00587 |
| ENSG00000235919 | DIABLO | 0.954239 | 0.005898 |
| ENSG00000179840 | CKLF-CMTM1 | 0.953991 | 0.005955 |
| ENSG00000251408 | FPGT | 0.953892 | 0.005973 |
| ENSG00000270177 | HIST1H2AK | 0.953786 | 0.005992 |
| ENSG00000224934 | PPIAL4G | 0.953227 | 0.006126 |
| ENSG00000231473 | DIABLO | 0.952335 | 0.006322 |
| ENSG00000263826 | HIST1H2AE | 0.952207 | 0.006346 |
| ENSG00000271869 | DIABLO | 0.951879 | 0.006421 |
| ENSG00000237803 | FPGT | 0.951802 | 0.006444 |
| ENSG00000234553 | FPGT | 0.951494 | 0.006505 |
| ENSG00000231970 | MRC1 | 0.950974 | 0.006647 |
| ENSG00000234028 | HIST1H3A | 0.950579 | 0.006714 |
| ENSG00000180422 | AKAP2 | 0.950545 | 0.006719 |
| ENSG00000270673 | HIST1H2AK | 0.950079 | 0.006814 |
| ENSG00000259895 | MRC1 | 0.949835 | 0.006854 |
| ENSG00000255062 | HIST1H3A | 0.948993 | 0.007057 |
| ENSG00000276744 | DIABLO | 0.947491 | 0.00743 |
| ENSG00000233030 | MGAM2 | 0.9472 | 0.007493 |
| ENSG00000273008 | HIST1H2AE | 0.946196 | 0.007757 |
| ENSG00000179840 | FAM47E | 0.944848 | 0.008094 |
| ENSG00000235919 | HIST1H3A | 0.94443 | 0.008225 |
| ENSG00000234553 | CKLF-CMTM1 | 0.942227 | 0.008812 |
| ENSG00000204283 | MLLT11 | 0.941655 | 0.008959 |
| ENSG00000270083 | DIABLO | 0.941087 | 0.009104 |
| ENSG00000237803 | MGAM2 | 0.940173 | 0.009359 |
| ENSG00000203327 | FPGT | 0.940117 | 0.009381 |
| ENSG00000230789 | MGAM2 | 0.939565 | 0.009515 |
| ENSG00000234553 | MGAM2 | 0.939087 | 0.009642 |
| ENSG00000268403 | HIST1H2AK | 0.939092 | 0.009642 |
| ENSG00000260877 | HIST1H2BG | 0.939039 | 0.009654 |
| ENSG00000236591 | FPGT | 0.938465 | 0.009803 |
| ENSG00000233030 | CKLF-CMTM1 | 0.938421 | 0.009816 |
| ENSG00000233993 | GPR162 | 0.937522 | 0.010031 |
| ENSG00000271869 | HIST1H3A | 0.936954 | 0.010222 |
| ENSG00000258602 | MRC1 | 0.936865 | 0.010249 |
| ENSG00000226756 | AKAP2 | 0.936727 | 0.010292 |
| ENSG00000279529 | DIABLO | 0.936155 | 0.010477 |
| ENSG00000179840 | MRC1 | 0.935942 | 0.010517 |
| ENSG00000179840 | LIPN | 0.935673 | 0.01059 |
| ENSG00000224934 | MRC1 | 0.935432 | 0.010659 |
| ENSG00000272843 | HIST1H3A | 0.935098 | 0.010778 |
| ENSG00000249378 | MRC1 | 0.933705 | 0.011213 |
| ENSG00000263884 | DIABLO | 0.93232 | 0.011638 |
| ENSG00000176236 | DXO | 0.930934 | 0.011969 |
| ENSG00000272256 | HIST1H3A | 0.929392 | 0.012415 |
| ENSG00000270673 | DIABLO | 0.927035 | 0.013175 |
| ENSG00000186056 | LAMB3 | 0.926783 | 0.013259 |
| ENSG00000221949 | HIST1H3A | 0.926556 | 0.013329 |
| ENSG00000260273 | HIST1H3A | 0.926467 | 0.013354 |
| ENSG00000186594 | GPR162 | 0.926155 | 0.013446 |
| ENSG00000180422 | DUSP28 | 0.926109 | 0.013464 |
| ENSG00000266680 | DIABLO | 0.925996 | 0.013496 |
| ENSG00000231473 | AKAP2 | 0.925605 | 0.013617 |
| ENSG00000273338 | HIST1H2AK | 0.92544 | 0.013666 |
| ENSG00000204283 | DXO | 0.924884 | 0.01385 |
| ENSG00000226308 | MRC1 | 0.924739 | 0.013895 |
| ENSG00000228786 | PPIAL4G | 0.924505 | 0.013967 |
| ENSG00000226308 | PPIAL4G | 0.923109 | 0.014427 |
| ENSG00000251408 | MRC1 | 0.922785 | 0.014526 |
| ENSG00000233611 | PPIAL4G | 0.921259 | 0.015032 |
| ENSG00000250410 | HIST1H2AE | 0.92097 | 0.015132 |
| ENSG00000272426 | HIST1H3A | 0.920863 | 0.015159 |
| ENSG00000230789 | MRC1 | 0.919178 | 0.015765 |
| ENSG00000236780 | MRC1 | 0.919177 | 0.015765 |
| ENSG00000236358 | CDRT4 | 0.919052 | 0.01579 |
| ENSG00000273117 | HIST1H3A | 0.918987 | 0.015805 |
| ENSG00000250410 | HIST1H3A | 0.918521 | 0.015956 |
| ENSG00000231948 | PPIAL4G | 0.917873 | 0.016175 |
| ENSG00000225979 | CKLF-CMTM1 | 0.916679 | 0.016594 |
| ENSG00000250410 | HIST1H2AK | 0.91575 | 0.016941 |
| ENSG00000228536 | MRC1 | 0.915665 | 0.016965 |
| ENSG00000275092 | HIST1H3A | 0.914519 | 0.017299 |
| ENSG00000272426 | DIABLO | 0.914052 | 0.017459 |
| ENSG00000275709 | HIST1H3A | 0.914012 | 0.017471 |
| ENSG00000236358 | MRC1 | 0.913234 | 0.017729 |
| ENSG00000260898 | HIST1H3A | 0.913119 | 0.017762 |
| ENSG00000270083 | HIST1H3A | 0.911542 | 0.018288 |
| ENSG00000180422 | DIABLO | 0.911519 | 0.018297 |
| ENSG00000228705 | PPIAL4G | 0.911031 | 0.018483 |
| ENSG00000251408 | CKLF-CMTM1 | 0.910775 | 0.018582 |
| ENSG00000271869 | HIST1H2AK | 0.910453 | 0.018692 |
| ENSG00000254791 | HIST1H3A | 0.910128 | 0.018831 |
| ENSG00000269220 | HIST1H3A | 0.909713 | 0.018997 |
| ENSG00000278330 | DIABLO | 0.909698 | 0.018998 |
| ENSG00000180422 | HIST1H3A | 0.909284 | 0.019185 |
| ENSG00000228786 | MRC1 | 0.908944 | 0.019291 |
| ENSG00000176236 | HLA-DOA | 0.908436 | 0.019489 |
| ENSG00000230789 | DNAJC25-GNG10 | 0.907734 | 0.019775 |
| ENSG00000248544 | MRC1 | 0.907736 | 0.019775 |
| ENSG00000235919 | HIST1H2AK | 0.907344 | 0.01994 |
| ENSG00000228536 | MGAM2 | 0.907064 | 0.020019 |
| ENSG00000260877 | HIST1H2AE | 0.906934 | 0.020067 |
| ENSG00000273117 | HIST1H2AK | 0.906566 | 0.02021 |
| ENSG00000231246 | HIST1H2AK | 0.906212 | 0.020332 |
| ENSG00000260101 | HIST1H3A | 0.906187 | 0.020335 |
| ENSG00000225979 | MRC1 | 0.905884 | 0.020456 |
| ENSG00000254551 | CKLF-CMTM1 | 0.905825 | 0.020484 |
| ENSG00000242828 | MGAM2 | 0.905449 | 0.02064 |
| ENSG00000278002 | DIABLO | 0.904343 | 0.021072 |
| ENSG00000272991 | HIST1H2BG | 0.904161 | 0.02113 |
| ENSG00000228889 | AKAP2 | 0.904059 | 0.021164 |
| ENSG00000260708 | HIST1H3A | 0.904031 | 0.021174 |
| ENSG00000272426 | HIST1H2AK | 0.902988 | 0.02152 |
| ENSG00000233143 | PPIAL4G | 0.902788 | 0.021575 |
| ENSG00000260708 | HIST1H2AK | 0.90257 | 0.021674 |
| ENSG00000263826 | HIST1H2BG | 0.902451 | 0.021726 |
| ENSG00000251408 | MGAM2 | 0.901829 | 0.021976 |
| ENSG00000225855 | MRC1 | 0.901523 | 0.022084 |
| ENSG00000231948 | FPGT | 0.90136 | 0.022153 |
| ENSG00000230789 | CKLF-CMTM1 | 0.900995 | 0.022295 |
| ENSG00000230309 | MRC1 | 0.900539 | 0.022522 |
| ENSG00000197210 | TSPOAP1 | 0.98495 | 0.000912 |
| ENSG00000228786 | HES2 | 0.989794 | 0.000462 |
| ENSG00000269318 | HES2 | 0.997664 | 1.20E-05 |
| ENSG00000255198 | JMJD6 | 0.991615 | 0.000316 |
| ENSG00000235919 | PPP1R15A | 0.994726 | 0.000107 |
| ENSG00000260898 | PPP1R15A | 0.984337 | 0.00098 |
| ENSG00000271869 | PPP1R15A | 0.989068 | 0.000521 |
| ENSG00000179840 | RNF24 | 0.98714 | 0.000699 |
| ENSG00000226756 | SEC14L5 | 0.985951 | 0.000806 |
| ENSG00000248629 | SEC14L5 | 0.987174 | 0.000699 |
| ENSG00000236591 | APLP1 | 0.990181 | 0.000432 |
| ENSG00000275092 | CD79A | 0.989325 | 0.0005 |
| ENSG00000286059 | GRM6 | 0.99424 | 0.000132 |
| ENSG00000263766 | GALNT3 | 0.989075 | 0.000521 |
| ENSG00000224789 | GBP1 | 0.988529 | 0.000566 |
| ENSG00000233029 | GBP1 | 0.985982 | 0.000804 |
| ENSG00000270030 | GBP1 | 0.992803 | 0.00022 |
| ENSG00000224789 | IFIT3 | 0.986167 | 0.000785 |
| ENSG00000233029 | IFIT3 | 0.996207 | 4.60E-05 |
| ENSG00000233029 | IFIT2 | 0.985668 | 0.000834 |
| ENSG00000260898 | PLEKHG1 | 0.987254 | 0.000697 |
| ENSG00000272356 | CCR2 | 0.984343 | 0.00098 |
| ENSG00000249378 | ITIH5 | 0.987823 | 0.00064 |
| ENSG00000272356 | ITIH5 | 0.986787 | 0.000735 |
| ENSG00000176236 | FOSB | 0.993532 | 0.000172 |
| ENSG00000230555 | FOSB | 0.987195 | 0.000699 |
| ENSG00000237054 | FOSB | 0.994562 | 0.000115 |
| ENSG00000245059 | FOSB | 0.987324 | 0.000691 |
| ENSG00000261202 | FOSB | 0.996709 | 3.20E-05 |
| ENSG00000268366 | FOSB | 0.994337 | 0.000128 |
| ENSG00000271821 | FOSB | 0.98659 | 0.00075 |
| ENSG00000275092 | FOSB | 0.984928 | 0.000913 |
| ENSG00000275437 | FOSB | 0.991221 | 0.000346 |
| ENSG00000261526 | ID1 | 0.989017 | 0.000525 |
| ENSG00000267858 | IQCN | 0.984469 | 0.000966 |
| ENSG00000268240 | IQCN | 0.99229 | 0.000258 |
| ENSG00000268658 | IQCN | 0.995315 | 8.10E-05 |
| MSTRG.18503 | IQCN | 0.993156 | 0.000195 |
| ENSG00000224789 | RTP4 | 0.991494 | 0.000327 |
| ENSG00000233029 | RTP4 | 0.992416 | 0.000249 |
| ENSG00000260507 | TLR4 | 0.984813 | 0.000921 |
| ENSG00000204283 | ANKRD42 | 0.986527 | 0.00075 |
| ENSG00000223653 | ANKRD42 | 0.989334 | 0.000499 |
| ENSG00000226828 | ANKRD42 | 0.987372 | 0.000685 |
| ENSG00000230555 | ANKRD42 | 0.994472 | 0.00012 |
| ENSG00000235527 | ANKRD42 | 0.989989 | 0.000445 |
| ENSG00000237054 | ANKRD42 | 0.993465 | 0.000175 |
| ENSG00000245059 | ANKRD42 | 0.992269 | 0.00026 |
| ENSG00000258413 | ANKRD42 | 0.985544 | 0.000844 |
| ENSG00000260273 | ANKRD42 | 0.987115 | 0.000699 |
| ENSG00000261202 | ANKRD42 | 0.989421 | 0.000494 |
| ENSG00000267858 | ANKRD42 | 0.986495 | 0.00075 |
| ENSG00000268366 | ANKRD42 | 0.995931 | 5.50E-05 |
| ENSG00000270175 | ANKRD42 | 0.989538 | 0.000484 |
| ENSG00000270361 | ANKRD42 | 0.985416 | 0.00086 |
| ENSG00000271821 | ANKRD42 | 0.990706 | 0.000389 |
| ENSG00000272256 | ANKRD42 | 0.984184 | 0.000995 |
| ENSG00000272735 | ANKRD42 | 0.986873 | 0.000727 |
| ENSG00000272843 | ANKRD42 | 0.986735 | 0.000739 |
| ENSG00000273702 | ANKRD42 | 0.987899 | 0.000632 |
| ENSG00000275092 | ANKRD42 | 0.984882 | 0.000916 |
| ENSG00000275198 | ANKRD42 | 0.991768 | 0.000306 |
| ENSG00000275437 | ANKRD42 | 0.986544 | 0.00075 |
| MSTRG.14063 | ANKRD42 | 0.98416 | 0.000995 |
| MSTRG.17935 | ANKRD42 | 0.986379 | 0.000763 |
| MSTRG.18503 | ANKRD42 | 0.989623 | 0.000476 |
| ENSG00000233029 | IFI44 | 0.985506 | 0.000849 |
| ENSG00000224789 | IFITM3 | 0.993754 | 0.000158 |
| ENSG00000233029 | IFITM3 | 0.99514 | 8.80E-05 |
| ENSG00000179840 | ATP6V1B2 | 0.998724 | 2.00E-06 |
| ENSG00000234553 | ATP6V1B2 | 0.985943 | 0.000806 |
| ENSG00000255198 | PTS | 0.993928 | 0.00015 |
| ENSG00000233029 | IFIT5 | 0.989334 | 0.000499 |
| ENSG00000179840 | ABCG1 | 0.986232 | 0.00078 |
| ENSG00000261451 | IL6R | 0.991344 | 0.00034 |
| MSTRG.2753 | IL6R | 0.987017 | 0.00071 |
| ENSG00000186056 | ARMC3 | 0.984765 | 0.000925 |
| ENSG00000221949 | LARGE2 | 0.989636 | 0.000476 |
| ENSG00000234028 | LARGE2 | 0.986129 | 0.00079 |
| ENSG00000268403 | LARGE2 | 0.987318 | 0.000691 |
| ENSG00000272843 | LARGE2 | 0.987789 | 0.000642 |
| ENSG00000278743 | LARGE2 | 0.984306 | 0.000982 |
| ENSG00000285871 | LARGE2 | 0.98693 | 0.000722 |
| ENSG00000231830 | COQ7 | 0.986208 | 0.000782 |
| ENSG00000260898 | COQ7 | 0.986231 | 0.00078 |
| ENSG00000267858 | COQ7 | 0.991749 | 0.000307 |
| ENSG00000275437 | COQ7 | 0.988889 | 0.000534 |
| MSTRG.14063 | COQ7 | 0.986579 | 0.00075 |
| MSTRG.18503 | COQ7 | 0.986002 | 0.000803 |
| ENSG00000224789 | BATF2 | 0.992622 | 0.000233 |
| ENSG00000233029 | BATF2 | 0.985126 | 0.000891 |
| ENSG00000176236 | FOS | 0.989396 | 0.000496 |
| ENSG00000230555 | FOS | 0.992445 | 0.000247 |
| ENSG00000237054 | FOS | 0.996628 | 3.40E-05 |
| ENSG00000245059 | FOS | 0.98601 | 0.000803 |
| ENSG00000261202 | FOS | 0.996147 | 4.80E-05 |
| ENSG00000268366 | FOS | 0.987999 | 0.000622 |
| ENSG00000270175 | FOS | 0.991141 | 0.00035 |
| ENSG00000270361 | FOS | 0.988225 | 0.000598 |
| ENSG00000271821 | FOS | 0.995016 | 9.10E-05 |
| ENSG00000272735 | FOS | 0.995742 | 6.20E-05 |
| ENSG00000275437 | FOS | 0.988792 | 0.000541 |
| MSTRG.14063 | FOS | 0.997169 | 2.10E-05 |
| MSTRG.18503 | FOS | 0.992322 | 0.000256 |
| ENSG00000226308 | PCDHB1 | 0.985237 | 0.000882 |
| ENSG00000276255 | PCDHB1 | 0.986741 | 0.000739 |
| ENSG00000278996 | RAPH1 | 0.987837 | 0.000639 |
| ENSG00000262714 | SULT1B1 | 0.989782 | 0.000462 |
| ENSG00000263766 | SULT1B1 | 0.987518 | 0.000671 |
| ENSG00000274536 | SULT1B1 | 0.985376 | 0.000865 |
| ENSG00000233029 | P2RY14 | 0.985617 | 0.00084 |
| ENSG00000253334 | ZFAND2A | 0.989258 | 0.000504 |
| ENSG00000231830 | SLC26A11 | 0.986531 | 0.00075 |
| ENSG00000286059 | TNFSF15 | 0.984917 | 0.000913 |
| ENSG00000260507 | ZNF716 | 0.990644 | 0.000394 |
| ENSG00000263766 | ZNF716 | 0.987652 | 0.000656 |
| ENSG00000270562 | ZNF716 | 0.991301 | 0.000341 |
| MSTRG.2753 | ZNF716 | 0.990168 | 0.000432 |
| ENSG00000270030 | FFAR3 | 0.988884 | 0.000534 |
| ENSG00000249378 | MYT1L | 0.990083 | 0.00044 |
| ENSG00000258602 | MYT1L | 0.988807 | 0.00054 |
| ENSG00000224789 | ISG15 | 0.987219 | 0.000699 |
| ENSG00000249378 | FAM47E | 0.985802 | 0.000818 |
| ENSG00000272356 | FAM47E | 0.988712 | 0.000548 |
| ENSG00000248629 | YRDC | 0.991134 | 0.00035 |
| ENSG00000261526 | TLE1 | 0.987797 | 0.000642 |
| ENSG00000270177 | HLA-DOA | 0.989995 | 0.000445 |
| ENSG00000270673 | HLA-DOA | 0.988553 | 0.000565 |
| ENSG00000271869 | HLA-DOA | 0.984712 | 0.000932 |
| ENSG00000254791 | DXO | 0.988467 | 0.000569 |
| ENSG00000271869 | DXO | 0.984348 | 0.00098 |
| ENSG00000230555 | C17orf107 | 0.986003 | 0.000803 |
| ENSG00000237054 | C17orf107 | 0.988987 | 0.000528 |
| ENSG00000270175 | C17orf107 | 0.988542 | 0.000565 |
| ENSG00000270361 | C17orf107 | 0.99119 | 0.000347 |
| ENSG00000272735 | C17orf107 | 0.989245 | 0.000504 |
| ENSG00000275092 | C17orf107 | 0.989806 | 0.000462 |
| MSTRG.18503 | C17orf107 | 0.984342 | 0.00098 |
| ENSG00000235527 | MLLT11 | 0.98528 | 0.000878 |
| ENSG00000259802 | MLLT11 | 0.993263 | 0.000191 |
| ENSG00000260475 | MLLT11 | 0.986204 | 0.000782 |
| ENSG00000269220 | MLLT11 | 0.991178 | 0.000347 |
| ENSG00000272843 | MLLT11 | 0.988504 | 0.000568 |
| ENSG00000278743 | MLLT11 | 0.990813 | 0.000381 |
| ENSG00000238058 | PPIAL4G | 0.986485 | 0.000751 |
| ENSG00000274536 | FPGT | 0.986813 | 0.000734 |

Table S5. The prediction of target genes of lncRNAs via cis-regulation or trans-regulation.

| lncRNA id | Target gene name |
| --- | --- |
| NONHSAT099604.2 | CSF2RA |
| NONHSAT193044.1 | EMD |
| NONHSAT102970.2 | CSF2RA |
| NONHSAT255073.1 | CSF2RA |
| NONHSAT237694.1 | CSF2RA |
| NONHSAT151269.1 | CSF2RA |
| NONHSAT229985.1 | CSF2RA |
| NONHSAT242206.1 | EMD |
| NONHSAT240984.1 | CSF2RA |
| NONHSAT002770.2 | CSF2RA |
| NONHSAT245985.1 | CSF2RA |
| XR_001753508.2 | CSF2RA |
| NONHSAT044063.2 | DERL1 |
| NONHSAT044063.2 | PHF20L1 |
| NONHSAT044063.2 | DNAJC25-GNG10 |
| NONHSAT044063.2 | HSPA5 |
| NONHSAT227353.1 | CSF2RA |
| NONHSAT044063.2 | SASH3 |
| ENST00000602507 | ARHGAP4 |
| NONHSAT231413.1 | EMD |
| NONHSAT226007.1 | EMD |
| NONHSAT244861.1 | ARHGAP4 |
| NONHSAT165394.1 | DERL1 |
| NONHSAT165394.1 | PHF20L1 |
| NONHSAT165394.1 | DNAJC25-GNG10 |
| NONHSAT165394.1 | HSPA5 |
| NONHSAT165394.1 | SASH3 |
| NR_109851.1 | EMD |
| NONHSAT239502.1 | CSF2RA |
| NONHSAT202191.1 | GTF2H2 |
| ENST00000501068 | BEND4 |
| ENST00000566847 | SDHA |
| ENST00000600534 | EIF4G1 |
| NONHSAT033792.2 | GTF2H2 |
| NONHSAT249123.1 | GTF2H2 |
| NONHSAT033797.2 | GTF2H2 |
| NONHSAT245361.1 | GTF2H2 |
| NR_145459.1 | LRPAP1 |
| NONHSAT102970.2 | GTF2H2 |
| NONHSAT255073.1 | GTF2H2 |
| NONHSAT237694.1 | GTF2H2 |
| NONHSAT240597.1 | GTF2H2 |
| NONHSAT242206.1 | EIF4G1 |
| NONHSAT151269.1 | GTF2H2 |
| NONHSAT242206.1 | GTF2H2 |
| NONHSAT237821.1 | GTF2H2 |
| ENST00000606164 | GTF2H2 |
| NONHSAT245776.1 | GTF2H2 |
| ENST00000641463 | GTF2H2 |
| NONHSAT240984.1 | GTF2H2 |
| NONHSAT217585.1 | GTF2H2 |
| NONHSAT002770.2 | GTF2H2 |
| ENST00000500989 | GTF2H2 |
| NONHSAT225049.1 | GTF2H2 |
| NONHSAT251895.1 | GTF2H2 |
| NONHSAT245985.1 | GTF2H2 |
| NONHSAT234736.1 | GTF2H2 |
| NONHSAT252157.1 | GTF2H2 |
| NONHSAT188509.1 | LRPAP1 |
| XR_936967.2 | EIF4G1 |
| NR_149152.1 | GTF2H2 |
| XR_001753508.2 | GTF2H2 |
| NONHSAT251227.1 | GTF2H2 |
| NONHSAT258633.1 | GTF2H2 |
| NONHSAT044063.2 | KLHL8 |
| NONHSAT227353.1 | GTF2H2 |
| NONHSAT044063.2 | GTF2H2 |
| NONHSAT044063.2 | PCDHB1 |
| NONHSAT231413.1 | GTF2H2 |
| NONHSAT227517.1 | GTF2H2 |
| NONHSAT227521.1 | GTF2H2 |
| NONHSAT231594.1 | GTF2H2 |
| NONHSAT235043.1 | GTF2H2 |
| NONHSAT239238.1 | GTF2H2 |
| NONHSAT248659.1 | GTF2H2 |
| NONHSAT235734.1 | GTF2H2 |
| NONHSAT244861.1 | GTF2H2 |
| NONHSAT200775.1 | GTF2H2 |
| NR_110473.1 | GTF2H2 |
| NONHSAT165394.1 | KLHL8 |
| NONHSAT165394.1 | GTF2H2 |
| NONHSAT248817.1 | GTF2H2 |
| NONHSAT165394.1 | PCDHB1 |
| NONHSAT239502.1 | GTF2H2 |
| NONHSAT099604.2 | POR |
| NONHSAT202191.1 | HIST1H2BN |
| NONHSAT249935.1 | HIST1H2BN |
| NONHSAT249123.1 | HIST1H2BN |
| NONHSAT249123.1 | BTBD9 |
| NONHSAT240433.1 | HIST1H2BN |
| NONHSAT102970.2 | HIST1H2BN |
| NONHSAT102970.2 | BTBD9 |
| NONHSAT237694.1 | HIST1H2BN |
| NONHSAT151269.1 | HIST1H2BN |
| NONHSAT229985.1 | HIST1H2BN |
| NONHSAT242206.1 | HIST1H2BN |
| NONHSAT229985.1 | BTBD9 |
| NONHSAT242206.1 | BTBD9 |
| NONHSAT256121.1 | BTBD9 |
| ENST00000605298 | HLA-B |
| ENST00000606164 | HIST1H2BG |
| ENST00000606164 | HIST1H2BN |
| ENST00000641463 | HIST1H2BN |
| NONHSAT002770.2 | HIST1H2BN |
| ENST00000615251 | HIST1H2BN |
| NONHSAT251895.1 | HIST1H2BN |
| NONHSAT234587.1 | ARID1B |
| NONHSAT234736.1 | HIST1H3A |
| NONHSAT252157.1 | HIST1H2BN |
| XR_001753508.2 | HIST1H3A |
| NR_149152.1 | HIST1H2BN |
| XR_001753508.2 | HIST1H2BN |
| NONHSAT190639.1 | HIST1H3A |
| NONHSAT251227.1 | HIST1H2BG |
| NONHSAT251227.1 | HIST1H2BN |
| NONHSAT044063.2 | HIST1H2BG |
| NONHSAT044063.2 | HIST1H2BN |
| ENST00000607434 | HIST1H2BN |
| NONHSAT044063.2 | HLA-DOA |
| NONHSAT231413.1 | HIST1H2BN |
| NONHSAT227517.1 | HIST1H2BN |
| NONHSAT227521.1 | HIST1H2BN |
| ENST00000623966 | BTBD9 |
| NONHSAT235043.1 | HIST1H2BG |
| NONHSAT231594.1 | HIST1H2BN |
| NONHSAT235043.1 | HIST1H2BN |
| NONHSAT239238.1 | HIST1H2BN |
| NONHSAT248659.1 | BTBD9 |
| NONHSAT244861.1 | HIST1H2BN |
| NR_110473.1 | HIST1H2BN |
| NONHSAT165394.1 | RIPOR2 |
| NONHSAT165394.1 | HIST1H2BG |
| NONHSAT165394.1 | HIST1H2BN |
| NONHSAT165394.1 | HLA-DOA |
| NONHSAT239502.1 | HIST1H2BN |
| ENST00000501068 | FOS |
| NONHSAT233228.1 | FOXN3 |
| NONHSAT193044.1 | RPS29 |
| NONHSAT193044.1 | SPTB |
| NONHSAT159210.1 | APBA2 |
| NONHSAT237821.1 | RPS29 |
| NONHSAT240984.1 | RPS29 |
| ENST00000412084 | FOS |
| NR_149152.1 | RPS29 |
| NONHSAT227353.1 | RPS29 |
| NONHSAT044063.2 | MYO5A |
| NONHSAT044063.2 | RAB27A |
| NONHSAT239238.1 | RPS29 |
| NONHSAT165394.1 | MYO5A |
| NONHSAT165394.1 | RAB27A |
| NONHSAT033737.2 | IGF1R |
| NONHSAT033737.2 | CIITA |
| NONHSAT099604.2 | RBM14 |
| NONHSAT099604.2 | NDUFV1 |
| NONHSAT099604.2 | MAP1LC3B |
| ENST00000606853 | ATP6V1D |
| ENST00000566847 | ANKRD42 |
| ENST00000501068 | WNK1 |
| ENST00000566847 | DIABLO |
| NONHSAT233228.1 | MIS18BP1 |
| NONHSAT245361.1 | GALC |
| NR_145459.1 | CCDC88B |
| NONHSAT255073.1 | CLEC4C |
| NONHSAT255073.1 | ZNF106 |
| NONHSAT240597.1 | RBM14 |
| ENST00000603538 | GALC |
| NONHSAT240597.1 | ZNF106 |
| NONHSAT229985.1 | ZNF106 |
| NONHSAT242206.1 | ZNF106 |
| NONHSAT237821.1 | ZNF106 |
| ENST00000602919 | SYCE1L |
| NONHSAT245776.1 | GALC |
| NONHSAT240984.1 | RBM14 |
| NONHSAT002770.2 | RBM14 |
| ENST00000412084 | GALC |
| NONHSAT002770.2 | ZNF106 |
| ENST00000412084 | IFT20 |
| NONHSAT251895.1 | ZNF106 |
| NONHSAT234587.1 | RASA3 |
| NONHSAT234736.1 | ZNF106 |
| NONHSAT188509.1 | CARMIL2 |
| NONHSAT235324.1 | SYCE1L |
| NONHSAT258633.1 | ZNF106 |
| NONHSAT044063.2 | CCDC88B |
| NONHSAT044063.2 | RBM14 |
| NONHSAT044063.2 | CLEC4C |
| NONHSAT044063.2 | CD69 |
| NONHSAT044063.2 | HMGB1 |
| NONHSAT044063.2 | EAPP |
| NONHSAT044063.2 | FAM177A1 |
| NONHSAT044063.2 | ATP6V1D |
| NONHSAT044063.2 | ZNF106 |
| NONHSAT044063.2 | THUMPD1 |
| NONHSAT044063.2 | MAP1LC3B |
| ENST00000602507 | MAP1LC3B |
| NONHSAT244861.1 | SRPRA |
| NONHSAT235734.1 | ZNF106 |
| NONHSAT200775.1 | ZNF106 |
| NONHSAT165394.1 | CCDC88B |
| NR_109851.1 | CCDC88B |
| NONHSAT165394.1 | RBM14 |
| NONHSAT165394.1 | CLEC4C |
| NONHSAT165394.1 | CD69 |
| NONHSAT165394.1 | HMGB1 |
| NONHSAT165394.1 | EAPP |
| NONHSAT165394.1 | FAM177A1 |
| NONHSAT165394.1 | ATP6V1D |
| NONHSAT165394.1 | CCNK |
| NONHSAT165394.1 | ZNF106 |
| NONHSAT248817.1 | ZNF106 |
| NONHSAT165394.1 | THUMPD1 |
| NONHSAT165394.1 | LYRM1 |
| NONHSAT165394.1 | PSMD7 |
| NR_109851.1 | SYCE1L |
| NONHSAT165394.1 | MAP1LC3B |
| NONHSAT033737.2 | ATP6V1D |
| ENST00000501068 | TMEM91 |
| ENST00000566847 | AC008755.1 |
| ENST00000501068 | AC008755.1 |
| ENST00000566847 | PPP1R15A |
| ENST00000566847 | PPP1R12C |
| NONHSAT229985.1 | FOSB |
| NR_125355.1 | EVPL |
| NONHSAT044063.2 | JMJD6 |
| NONHSAT044063.2 | THOP1 |
| ENST00000565797 | THOP1 |
| ENST00000565797 | JUND |
| NONHSAT044063.2 | ZNF470 |
| NONHSAT244861.1 | PPP1R15A |
| NONHSAT165394.1 | JMJD6 |
| NONHSAT165394.1 | FECH |
| NONHSAT165394.1 | THOP1 |
| NONHSAT165394.1 | ZNF226 |
| NONHSAT233228.1 | SH3BP2 |
| NONHSAT233228.1 | RBM47 |
| NONHSAT249935.1 | STX11 |
| ENST00000501068 | CHST12 |
| NONHSAT249123.1 | STX11 |
| NONHSAT240597.1 | STX11 |
| NONHSAT151269.1 | STX11 |
| NONHSAT242206.1 | STX11 |
| NONHSAT217585.1 | STX11 |
| NONHSAT002770.2 | STX11 |
| NONHSAT251895.1 | STX11 |
| NONHSAT234587.1 | UBE2H |
| NONHSAT246144.1 | PPP1R2 |
| NONHSAT258633.1 | STX11 |
| NONHSAT044063.2 | HMGCS1 |
| ENST00000602507 | CDK19 |
| NONHSAT044063.2 | ZKSCAN1 |
| NONHSAT044063.2 | ZC3HAV1 |
| NONHSAT044063.2 | SLC25A37 |
| NONHSAT044063.2 | BNIP3L |
| NONHSAT244832.1 | LAP3 |
| NONHSAT231594.1 | STX11 |
| NONHSAT239238.1 | STX11 |
| NONHSAT248659.1 | STX11 |
| NONHSAT200775.1 | STX11 |
| NR_110473.1 | STX11 |
| NONHSAT165394.1 | PPP1R2 |
| NONHSAT165394.1 | SH3BP2 |
| NONHSAT165394.1 | RBM47 |
| NONHSAT165394.1 | HMGCS1 |
| NONHSAT165394.1 | CDK19 |
| NONHSAT165394.1 | BCLAF1 |
| NONHSAT248817.1 | STX11 |
| NONHSAT165394.1 | SBDS |
| NONHSAT165394.1 | ZKSCAN1 |
| NONHSAT165394.1 | ZC3HAV1 |
| NONHSAT165394.1 | SLC25A37 |
| NONHSAT229095.1 | TMC8 |
| NONHSAT229095.1 | BCL6 |
| ENST00000566847 | P4HB |
| NONHSAT233228.1 | PREX1 |
| ENST00000501068 | TSC22D2 |
| NONHSAT245361.1 | TMC8 |
| NONHSAT240597.1 | TMC8 |
| NONHSAT151269.1 | TMC8 |
| ENST00000602919 | TMC8 |
| NONHSAT002770.2 | TMC8 |
| ENST00000412084 | U2AF1 |
| ENST00000412084 | TYMP |
| ENST00000602461 | JMJD6 |
| ENST00000408887 | CIRBP |
| ENST00000602461 | PIK3IP1 |
| ENST00000602461 | TSC22D2 |
| NONHSAT044063.2 | MAP2K6 |
| NONHSAT044063.2 | IQCN |
| ENST00000565797 | IQCN |
| NONHSAT044063.2 | UBXN4 |
| NONHSAT044063.2 | STK17B |
| NONHSAT044063.2 | SF3A1 |
| ENST00000602507 | PIK3IP1 |
| NONHSAT044063.2 | ZBTB11 |
| ENST00000602507 | TSC22D2 |
| NONHSAT244832.1 | PIK3IP1 |
| NONHSAT235734.1 | SPOP |
| NONHSAT200775.1 | TMC8 |
| NONHSAT165394.1 | MAP2K6 |
| NONHSAT165394.1 | TMC8 |
| NONHSAT248817.1 | TMC8 |
| NONHSAT165394.1 | IQCN |
| NONHSAT165394.1 | UBXN4 |
| NONHSAT165394.1 | STK17B |
| NONHSAT165394.1 | SF3A1 |
| NONHSAT165394.1 | ZBTB11 |
| NONHSAT033737.2 | STK16 |
| ENST00000501068 | WASHC2A |
| NONHSAT249123.1 | HES2 |
| ENST00000600534 | JUN |
| NONHSAT240433.1 | HES2 |
| NONHSAT102970.2 | HES2 |
| NONHSAT255073.1 | HES2 |
| NONHSAT240597.1 | HES2 |
| NONHSAT151269.1 | HES2 |
| NONHSAT229985.1 | HES2 |
| NONHSAT242206.1 | HES2 |
| NR_125355.1 | HES2 |
| ENST00000606164 | HES2 |
| NONHSAT240984.1 | HES2 |
| NONHSAT217585.1 | HES2 |
| NONHSAT002770.2 | HES2 |
| NONHSAT246933.1 | HES2 |
| NONHSAT251895.1 | HES2 |
| NONHSAT234587.1 | C1orf159 |
| NONHSAT252157.1 | HES2 |
| NR_038337.2 | HES2 |
| NR_149152.1 | HES2 |
| NONHSAT251227.1 | HES2 |
| NONHSAT227353.1 | HES2 |
| NONHSAT044063.2 | HES2 |
| ENST00000607434 | HES2 |
| ENST00000602507 | HES2 |
| NONHSAT044063.2 | GNAI3 |
| ENST00000565797 | TAGLN2 |
| NONHSAT235451.1 | HES2 |
| NONHSAT235043.1 | HES2 |
| NONHSAT248659.1 | HES2 |
| NONHSAT244832.1 | MRPL55 |
| NONHSAT244861.1 | HES2 |
| NONHSAT200775.1 | HES2 |
| XR_001755978.1 | AMPD2 |
| NONHSAT165394.1 | HES2 |
| NONHSAT248817.1 | HES2 |
| NONHSAT165394.1 | GNAI3 |
| NONHSAT239502.1 | HES2 |
| ENST00000606853 | AC003665.1 |
| ENST00000566847 | KAT8 |
| ENST00000501068 | ACSF3 |
| NONHSAT233228.1 | ATP2A3 |
| NONHSAT233228.1 | AC003665.1 |
| ENST00000596206 | ADGRG1 |
| NONHSAT193044.1 | C17orf107 |
| ENST00000602461 | NLRC5 |
| ENST00000602461 | ATP2A3 |
| ENST00000602507 | NLRC5 |
| ENST00000602507 | ATP2A3 |
| ENST00000602507 | C17orf107 |
| NONHSAT044063.2 | SLFN13 |
| NONHSAT227517.1 | ATP2A3 |
| NONHSAT244861.1 | NLRC5 |
| NONHSAT165394.1 | COQ7 |
| NONHSAT165394.1 | SLFN13 |
| ENST00000566847 | TNS1 |
| ENST00000574306 | TNS1 |
| NONHSAT044063.2 | TRAPPC2B |
| NONHSAT044063.2 | GPR155 |
| NONHSAT044063.2 | AP1S3 |
| NONHSAT235451.1 | NCL |
| NONHSAT165394.1 | TRAPPC2B |
| NONHSAT165394.1 | GPR155 |
| NONHSAT165394.1 | AP1S3 |
| ENST00000606853 | ESYT2 |
| NONHSAT249123.1 | DOCK4 |
| NONHSAT249123.1 | GINS4 |
| ENST00000607315 | AKAP2 |
| NONHSAT255073.1 | DOCK4 |
| NONHSAT102970.2 | GINS4 |
| NONHSAT240597.1 | GINS4 |
| NONHSAT229985.1 | DOCK4 |
| NONHSAT237821.1 | DOCK4 |
| ENST00000606164 | DOCK4 |
| ENST00000606164 | GINS4 |
| NONHSAT217585.1 | IDO1 |
| ENST00000641463 | GINS4 |
| NONHSAT240984.1 | GINS4 |
| NONHSAT217585.1 | GINS4 |
| NONHSAT246933.1 | DOCK4 |
| NONHSAT002770.2 | GINS4 |
| NONHSAT252157.1 | GINS4 |
| NR_149152.1 | DOCK4 |
| XR_001753508.2 | DOCK4 |
| ENST00000602461 | ZNF746 |
| NR_149152.1 | GINS4 |
| NONHSAT251227.1 | GINS4 |
| NONHSAT044063.2 | DOCK4 |
| NONHSAT044063.2 | ESYT2 |
| NONHSAT044063.2 | IDO1 |
| NONHSAT227353.1 | GINS4 |
| NONHSAT044063.2 | GINS4 |
| ENST00000607434 | GINS4 |
| NONHSAT235451.1 | GINS4 |
| NONHSAT231594.1 | DOCK4 |
| NONHSAT248659.1 | DOCK4 |
| NONHSAT235043.1 | GINS4 |
| NONHSAT235734.1 | GINS4 |
| NONHSAT244861.1 | GINS4 |
| NR_110473.1 | GINS4 |
| NONHSAT165394.1 | DOCK4 |
| NONHSAT165394.1 | ESYT2 |
| NONHSAT165394.1 | IDO1 |
| NONHSAT165394.1 | GINS4 |
| NONHSAT239502.1 | GINS4 |
| ENST00000566847 | RNH1 |
| NONHSAT233228.1 | TOLLIP |
| NONHSAT255073.1 | C11orf54 |
| NONHSAT229985.1 | C11orf54 |
| NONHSAT242206.1 | C11orf54 |
| NONHSAT256121.1 | C11orf54 |
| NONHSAT237821.1 | C11orf54 |
| NONHSAT238009.1 | CAPN1 |
| NONHSAT002770.2 | C11orf54 |
| NR_126534.1 | TCIRG1 |
| NONHSAT044063.2 | IFIT3 |
| ENST00000602507 | PIDD1 |
| ENST00000602507 | ILK |
| NONHSAT044063.2 | KCNE3 |
| NONHSAT231413.1 | C11orf54 |
| NONHSAT226007.1 | C11orf54 |
| NONHSAT248659.1 | C11orf54 |
| NONHSAT165394.1 | IFIT3 |
| NONHSAT165394.1 | KCNE3 |
| NONHSAT165394.1 | C11orf54 |
| NONHSAT233228.1 | FBXW8 |
| NONHSAT245361.1 | CHD8 |
| ENST00000412084 | UBC |
| ENST00000602461 | KRT73 |
| NONHSAT044063.2 | FGD4 |
| NONHSAT044063.2 | IRAK4 |
| ENST00000602507 | KRT73 |
| ENST00000565797 | FBXW8 |
| ENST00000602507 | HIP1R |
| NONHSAT044063.2 | CHD8 |
| NONHSAT165394.1 | FGD4 |
| NONHSAT165394.1 | IRAK4 |
| NONHSAT165394.1 | SCAF11 |
| NONHSAT165394.1 | SLC4A8 |
| NONHSAT165394.1 | CHD8 |
| NONHSAT033737.2 | IRAK4 |
| NONHSAT099604.2 | PTGDS |
| ENST00000606853 | NOTCH1 |
| ENST00000501068 | ZNF683 |
| ENST00000566847 | PLK3 |
| ENST00000501068 | PLK3 |
| NONHSAT233228.1 | IRF2BP2 |
| ENST00000600534 | ZC3H12A |
| NONHSAT240433.1 | CTBS |
| NONHSAT151269.1 | PLK3 |
| NONHSAT151269.1 | CTBS |
| NONHSAT229985.1 | CTBS |
| NONHSAT256121.1 | CTBS |
| NONHSAT002770.2 | CTBS |
| NONHSAT251895.1 | PLK3 |
| NONHSAT234736.1 | CTBS |
| ENST00000602461 | DDIT4 |
| ENST00000602461 | ITPRIP |
| ENST00000565797 | AKAP17A |
| NONHSAT044063.2 | BEND2 |
| NONHSAT044063.2 | XIAP |
| NONHSAT044063.2 | EIF3I |
| NONHSAT044063.2 | PLK3 |
| NONHSAT044063.2 | AIM2 |
| NONHSAT044063.2 | ECD |
| NONHSAT044063.2 | ITPRIP |
| NONHSAT231594.1 | PLK3 |
| NONHSAT244861.1 | CTBS |
| NONHSAT165394.1 | BEND2 |
| NONHSAT165394.1 | XIAP |
| NONHSAT165394.1 | EIF3I |
| NONHSAT165394.1 | PLK3 |
| NONHSAT165394.1 | AIM2 |
| NONHSAT165394.1 | ECD |
| NONHSAT165394.1 | ITPRIP |
| NR_152571.1 | SIRPB1 |
| NONHSAT099604.2 | NCAPH2 |
| NONHSAT233228.1 | IQSEC1 |
| ENST00000606596 | ATF4 |
| ENST00000622671 | XBP1 |
| NONHSAT234587.1 | RNF24 |
| NONHSAT044063.2 | RNF24 |
| NONHSAT044063.2 | DSN1 |
| NONHSAT044063.2 | ZNFX1 |
| NONHSAT044063.2 | MX2 |
| NONHSAT165394.1 | RNF24 |
| NONHSAT165394.1 | DSN1 |
| NONHSAT165394.1 | ZNFX1 |
| NONHSAT165394.1 | MX2 |
| NONHSAT165394.1 | FAM118A |
| MSTRG.21193.1 | MZT2A |
| MSTRG.11401.1 | TRAPPC2B |
| MSTRG.11401.1 | GPR155 |
| MSTRG.11401.1 | AP1S3 |
| MSTRG.13467.1 | GLYR1 |
| MSTRG.21193.1 | APBA2 |
| MSTRG.21193.1 | NUDT16L1 |
| MSTRG.18433.7 | TSC2 |
| MSTRG.19902.1 | CRIP1 |
| MSTRG.11401.1 | MYO5A |
| MSTRG.11401.1 | RAB27A |
| MSTRG.11401.1 | SEC14L5 |
| MSTRG.13467.1 | KAT8 |
| MSTRG.13467.1 | MAPK7 |
| MSTRG.13476.2 | C17orf107 |
| MSTRG.21193.1 | THRA |
| MSTRG.5643.1 | C17orf107 |
| MSTRG.19902.1 | ATXN2L |
| MSTRG.19902.1 | C17orf107 |
| MSTRG.20264.2 | C17orf107 |
| MSTRG.11401.1 | COQ7 |
| MSTRG.11401.1 | SLFN13 |
| MSTRG.19902.1 | HIP1R |
| MSTRG.19902.1 | REM2 |
| MSTRG.11401.1 | FGD4 |
| MSTRG.11401.1 | IRAK4 |
| MSTRG.11401.1 | SCAF11 |
| MSTRG.555.1 | GTF2H2 |
| MSTRG.13467.1 | EIF4G1 |
| MSTRG.13467.1 | MFSD10 |
| MSTRG.13476.2 | BEND4 |
| MSTRG.13476.2 | SDHA |
| MSTRG.13476.2 | HNRNPH1 |
| MSTRG.15865.4 | GTF2H2 |
| MSTRG.16195.4 | GTF2H2 |
| MSTRG.5643.1 | DGKQ |
| MSTRG.17687.2 | GTF2H2 |
| MSTRG.18433.7 | GTF2H2 |
| MSTRG.11401.1 | LRPAP1 |
| MSTRG.11401.1 | KLHL8 |
| MSTRG.11401.1 | GTF2H2 |
| MSTRG.11401.1 | PCDHB1 |
| MSTRG.13467.1 | EMD |
| MSTRG.18433.7 | TOR2A |
| MSTRG.19902.1 | ARHGAP4 |
| MSTRG.11401.1 | PHF20L1 |
| MSTRG.11401.1 | DNAJC25-GNG10 |
| MSTRG.11401.1 | SASH3 |
| MSTRG.13467.1 | NOTCH1 |
| MSTRG.13467.1 | NSMF |
| MSTRG.13467.1 | ADAM15 |
| MSTRG.1071.2 | NSMF |
| MSTRG.13476.2 | MAGEE1 |
| MSTRG.13476.2 | ZC3H12A |
| MSTRG.5643.1 | AKAP17A |
| MSTRG.6104.4 | CDK16 |
| MSTRG.19902.1 | NOTCH1 |
| MSTRG.19902.1 | PTGDS |
| MSTRG.19902.1 | TSPYL2 |
| MSTRG.19902.1 | RRP12 |
| MSTRG.19902.1 | NXF1 |
| MSTRG.20264.2 | CXorf40A |
| MSTRG.20264.2 | ZC3H12A |
| MSTRG.11401.1 | XIAP |
| MSTRG.11401.1 | EIF3I |
| MSTRG.11401.1 | PLK3 |
| MSTRG.21193.1 | TNKS |
| MSTRG.15865.4 | GINS4 |
| MSTRG.16195.4 | DOCK4 |
| MSTRG.16195.4 | GINS4 |
| MSTRG.5643.1 | MCM7 |
| MSTRG.5643.1 | AKNA |
| MSTRG.18433.7 | GINS4 |
| MSTRG.19902.1 | AKNA |
| MSTRG.20264.2 | ESYT2 |
| MSTRG.11401.1 | ESYT2 |
| MSTRG.11401.1 | IDO1 |
| MSTRG.21193.1 | FUS |
| MSTRG.17935.2 | PATL2 |
| MSTRG.19902.1 | CCDC88B |
| MSTRG.19902.1 | RBM14 |
| MSTRG.19902.1 | HMGB1 |
| MSTRG.20264.2 | WNK1 |
| MSTRG.20264.2 | ULK3 |
| MSTRG.11401.1 | STX5 |
| MSTRG.11401.1 | CCDC88B |
| MSTRG.11401.1 | CLEC4C |
| MSTRG.11401.1 | CD69 |
| MSTRG.11401.1 | HMGB1 |
| MSTRG.11401.1 | FAM177A1 |
| MSTRG.11401.1 | CCNK |
| MSTRG.11401.1 | ZNF106 |
| MSTRG.11401.1 | THUMPD1 |
| MSTRG.11401.1 | LYRM1 |
| MSTRG.11401.1 | MAP1LC3B |
| MSTRG.13476.2 | SMOX |
| MSTRG.13476.2 | XRCC6 |
| MSTRG.5643.1 | SEC14L2 |
| MSTRG.5643.1 | XRCC6 |
| MSTRG.20264.2 | ACSS1 |
| MSTRG.11401.1 | DSN1 |
| MSTRG.11401.1 | ZNFX1 |
| MSTRG.11401.1 | MX2 |
| MSTRG.555.1 | HIST1H2BN |
| MSTRG.13467.1 | TMEM63B |
| MSTRG.21193.1 | TMEM63B |
| MSTRG.16195.4 | HIST1H2BN |
| MSTRG.17687.2 | HIST1H2BN |
| MSTRG.18433.7 | HIST1H2BN |
| MSTRG.6104.4 | TMEM63B |
| MSTRG.8516.8 | BTBD9 |
| MSTRG.8516.11 | BTBD9 |
| MSTRG.11401.1 | RIPOR2 |
| MSTRG.11401.1 | HIST1H2BG |
| MSTRG.11401.1 | HIST1H2BN |
| MSTRG.13476.2 | YTHDF1 |
| MSTRG.13476.2 | BCL6 |
| MSTRG.6104.4 | SEC14L1 |
| MSTRG.20264.2 | IQCN |
| MSTRG.11401.1 | MAP2K6 |
| MSTRG.11401.1 | JMJD6 |
| MSTRG.11401.1 | TMC8 |
| MSTRG.11401.1 | IQCN |
| MSTRG.11401.1 | SF3A1 |
| MSTRG.11401.1 | ZBTB11 |
| MSTRG.13467.1 | TTYH2 |
| MSTRG.13467.1 | RPL28 |
| MSTRG.13476.2 | ZNF236 |
| MSTRG.13476.2 | FOSB |
| MSTRG.13476.2 | PPP1R15A |
| MSTRG.18433.7 | EVPL |
| MSTRG.20264.2 | ARHGEF1 |
| MSTRG.11401.1 | THOP1 |
| MSTRG.11401.1 | ZNF226 |
| MSTRG.11401.1 | FOSB |
| MSTRG.11401.1 | ZNF470 |
| MSTRG.13467.1 | TOLLIP |
| MSTRG.21193.1 | ST3GAL4 |
| MSTRG.14213.1 | TCIRG1 |
| MSTRG.15865.4 | C11orf54 |
| MSTRG.18433.7 | TOLLIP |
| MSTRG.8516.8 | CAPN1 |
| MSTRG.8516.8 | TCIRG1 |
| MSTRG.19902.1 | TOLLIP |
| MSTRG.8516.11 | CAPN1 |
| MSTRG.8516.11 | TCIRG1 |
| MSTRG.11401.1 | ANKRD22 |
| MSTRG.11401.1 | IFIT3 |
| MSTRG.11401.1 | TMEM258 |
| MSTRG.11401.1 | KCNE3 |
| MSTRG.11401.1 | C11orf54 |
| MSTRG.13467.1 | SH3BP2 |
| MSTRG.5643.1 | TIAM2 |
| MSTRG.20264.2 | CHST12 |
| MSTRG.11401.1 | PPP1R2 |
| MSTRG.11401.1 | NCBP2 |
| MSTRG.11401.1 | CDK19 |
| MSTRG.11401.1 | SBDS |
| MSTRG.11401.1 | ZC3HAV1 |
| MSTRG.11401.1 | SLC25A37 |
| MSTRG.11401.1 | BNIP3L |
| MSTRG.11401.1 | KLF10 |
| MSTRG.15865.4 | HES2 |
| MSTRG.17687.2 | HES2 |
| MSTRG.5643.1 | JUN |
| MSTRG.17935.2 | HES2 |
| MSTRG.11401.1 | HES2 |
| MSTRG.11401.1 | GNAI3 |
| MSTRG.10574.2 | SAMD1 |
| MSTRG.17687.2 | AL135905.2 |
| MSTRG.13921.1 | PIK3IP1 |
| MSTRG.21623.8 | ALG13 |
| MSTRG.10574.2 | PRKACA |
| MSTRG.19614.6 | RPL30 |
| MSTRG.5954.1 | NDRG2 |
| MSTRG.8468.1 | ANKRD11 |
| MSTRG.10574.2 | CCDC130 |
| MSTRG.2259.8 | IDI1 |
| MSTRG.6104.4 | NFKBIA |
| MSTRG.10574.2 | PODNL1 |
| MSTRG.18433.7 | H2AFV |
| MSTRG.20264.2 | CARD19 |
| MSTRG.7677.1 | MGRN1 |
| MSTRG.5954.1 | ARHGEF40 |
| MSTRG.15699.39 | SEC31A |
| MSTRG.16807.2 | NDUFA2 |
| MSTRG.13701.12 | BID |
| MSTRG.10574.2 | MISP3 |
| MSTRG.10794.2 | ZNF257 |
| MSTRG.11873.4 | RAB1A |
| MSTRG.8660.9 | EIF4A1 |
| MSTRG.11401.1 | ZNF274 |
| MSTRG.8516.11 | INPP5K |
| MSTRG.8516.8 | INPP5K |
| MSTRG.10574.2 | NANOS3 |
| MSTRG.10574.2 | C19orf67 |
| MSTRG.16195.4 | SUB1 |
| MSTRG.12046.3 | GNLY |
| MSTRG.10574.2 | CC2D1A |
| MSTRG.13921.1 | LIMK2 |
| MSTRG.13644.4 | U2AF1 |
| MSTRG.10574.2 | C19orf53 |
| MSTRG.555.1 | C1orf122 |
| MSTRG.3274.1 | ADM |
| MSTRG.1841.1 | RGS1 |
| MSTRG.16807.2 | IK |
| MSTRG.17687.2 | PTP4A1 |
| MSTRG.10574.2 | DCAF15 |
| MSTRG.1071.2 | TAF13 |
| MSTRG.10574.2 | ADGRL1 |
| MSTRG.10574.2 | C19orf57 |
| MSTRG.15865.4 | PDE5A |
| MSTRG.3274.1 | SBF2 |
| MSTRG.14225.32 | THUMPD3 |
| MSTRG.18161.5 | ZFAND2A |
| MSTRG.10574.2 | ZSWIM4 |
| MSTRG.10574.2 | RFX1 |
| MSTRG.16297.1 | ANKRD55 |
| MSTRG.10574.2 | ASF1B |
| MSTRG.14246.1 | FANCD2 |
| MSTRG.16807.2 | TMCO6 |
| MSTRG.656.1 | ARMH1 |
| MSTRG.655.8 | ARMH1 |
| MSTRG.14213.1 | BHLHE40 |
| MSTRG.2903.1 | C10orf95 |
| MSTRG.17295.19 | RIPOR2 |
| MSTRG.5643.1 | RGCC |
| MSTRG.10574.2 | PALM3 |
| MSTRG.19445.1 | MCMDC2 |
| MSTRG.3274.1 | AMPD3 |
| MSTRG.10574.2 | IL27RA |
| MSTRG.20264.2 | NINJ1 |
| MSTRG.10574.2 | MRI1 |
| MSTRG.555.1 | YRDC |
| ENST00000577781 | EIF4A2 |
| NONHSAT159210.1 | VEGFB |
| ENST00000617588 | SIN3A |
| NONHSAT156100.1 | PDCD4 |
| NONHSAT156933.1 | HNRNPF |
| NONHSAT231435.1 | POC1B-GALNT4 |
| NONHSAT152123.1 | HIPK1 |
| NONHSAT227036.1 | HIPK1 |
| ENST00000584934 | AL135905.2 |
| NONHSAT185450.1 | EPB41L5 |
| NR_125355.1 | ANKRD42 |
| NONHSAT231435.1 | POC1B |
| NR_126035.1 | C12orf57 |
| NONHSAT161885.1 | ATF7IP |
| NONHSAT179754.1 | HNRNPUL1 |
| NONHSAT255070.1 | RB1CC1 |
| ENST00000610813 | KAT8 |
| NONHSAT179525.1 | RPL18A |
| NONHSAT086678.2 | CSNK1E |
| NONHSAT238009.1 | KPNB1 |
| NONHSAT217397.1 | RPL30 |
| ENST00000548760 | TMPO |
| NONHSAT097602.2 | UBE2D3 |
| NONHSAT239502.1 | SIGLEC10 |
| NONHSAT204279.1 | SMN2 |
| NONHSAT240984.1 | CLASP1 |
| NONHSAT054356.2 | CDK5RAP3 |
| NONHSAT246959.1 | CCNL1 |
| NONHSAT245985.1 | CCNL1 |
| NONHSAT173932.1 | CCDC102A |
| NONHSAT079576.2 | MYL9 |
| NR_104448.1 | SLC22A23 |
| ENST00000563592 | ADPGK |
| NONHSAT231594.1 | CORO1C |
| XR_002957397.1 | NCAPD2 |
| NONHSAT183063.1 | PPIG |
| NONHSAT160123.1 | SERGEF |
| NONHSAT254883.1 | ASAH1 |
| NONHSAT173188.1 | CBFB |
| NONHSAT248402.1 | SEC31A |
| ENST00000504718 | SEC31A |
| ENST00000504792 | SEC31A |
| NONHSAT247597.1 | SEC31A |
| ENST00000608395 | MBNL1 |
| NONHSAT253549.1 | AC000120.3 |
| NONHSAT200572.1 | LRBA |
| NONHSAT240180.1 | MYADM |
| NR_027873.1 | PAXBP1 |
| NR_146339.1 | CATSPER2 |
| ENST00000606596 | SARAF |
| NONHSAT189609.1 | RBM39 |
| ENST00000590717 | GAPDHS |
| NONHSAT079576.2 | TGIF2 |
| NONHSAT086730.2 | SUN2 |
| NONHSAT258675.1 | NUDT10 |
| NR_148678.1 | KDM4C |
| NONHSAT053824.2 | NBR1 |
| NONHSAT237917.1 | NBR1 |
| ENST00000560518 | GNB5 |
| NONHSAT230573.1 | ALDH3B1 |
| NONHSAT238330.1 | DLGAP1 |
| ENST00000640557 | CSRNP1 |
| ENST00000563278 | MAN2C1 |
| NONHSAT235734.1 | ALDH1A3 |
| NONHSAT231413.1 | DUSP6 |
| NONHSAT237694.1 | C17orf49 |
| NONHSAT200188.1 | PPM1K |
| NONHSAT227517.1 | CD46 |
| NONHSAT227516.1 | CD46 |
| NONHSAT227521.1 | CD46 |
| NONHSAT257506.1 | ZFAND5 |
| NR_131765.1 | TOGARAM1 |
| NONHSAT212135.1 | SUMF2 |
| XR_001753508.2 | MBP |
| NONHSAT086726.2 | SUN2 |
| NR_126534.1 | ZCCHC2 |
| ENST00000563151 | TK2 |
| XR_946984.2 | EFHD2 |
| ENST00000424245 | RBM23 |
| NONHSAT212780.1 | LUC7L2 |
| NR_104485.1 | RNPS1 |
| NONHSAT160123.1 | KCNC1 |
| NONHSAT237332.1 | RNF43 |
| NR_147047.1 | AC139768.1 |
| NR_147049.1 | AC139768.1 |
| NONHSAT225206.1 | ZNF326 |
| NONHSAT086730.2 | GTPBP1 |
| NONHSAT180222.1 | JAK3 |
| NONHSAT225182.1 | BCL10 |
| NONHSAT222719.1 | TSPYL2 |
| ENST00000624760 | RPP38 |
| NONHSAT251239.1 | HMGN3 |
| ENST00000607222 | MIB2 |
| ENST00000607434 | REV3L |
| NONHSAT252157.1 | TRIM39 |
| NR_038315.1 | ITGB2 |
| ENST00000596206 | ZNF143 |
| NONHSAT235451.1 | ARIH1 |
| ENST00000565181 | ARIH1 |
| NONHSAT192306.1 | MCM5 |
| NR_015431.2 | MKLN1 |
| NR_109851.1 | MKLN1 |
| NONHSAT228238.1 | SGMS1 |
| NONHSAT195547.1 | OSBPL10 |
| NONHSAT179463.1 | CALR |
| NONHSAT151269.1 | ELOA |
| NONHSAT173634.1 | MARF1 |
| NR_104487.1 | ZDHHC20 |
| ENST00000600534 | MZF1 |
| NONHSAT239396.1 | POU2F2 |
| NONHSAT240016.1 | SERTAD3 |
| NR_104487.1 | ZDHHC20-IT1 |
| NR_104448.1 | PSMG4 |
| NONHSAT240597.1 | PPP4R3B |
| NR_003109.1 | TRNAU1AP |
| ENST00000481220 | TRNAU1AP |
| NONHSAT237694.1 | RNASEK |
| ENST00000608025 | TRABD |
| ENST00000609885 | PRMT5 |
| ENST00000424245 | PRMT5 |
| NONHSAT083229.2 | BID |
| NR_131765.1 | KLHL28 |
| NONHSAT256574.1 | B4GALT1 |
| NONHSAT202191.1 | SRD5A1 |
| NONHSAT195845.1 | SLC25A26 |
| NONHSAT001280.2 | EMC1 |
| NONHSAT252991.1 | DDX39B |
| ENST00000580420 | TRAPPC8 |
| ENST00000608799 | POM121 |
| NONHSAT253549.1 | CYP51A1 |
| NONHSAT225182.1 | DDAH1 |
| ENST00000371417 | ARRDC1 |
| NONHSAT149754.1 | PDE4DIP |
| NONHSAT030431.2 | CHST11 |
| NONHSAT237428.1 | HELZ |
| NONHSAT175200.1 | SMARCE1 |
| XR_001746653.2 | AL162231.1 |
| ENST00000608266 | FMC1-LUC7L2 |
| NONHSAT212780.1 | FMC1-LUC7L2 |
| ENST00000501068 | AC092718.3 |
| NONHSAT079576.2 | TGIF2-RAB5IF |
| ENST00000580891 | RALBP1 |
| NONHSAT196311.1 | SLC35G2 |
| ENST00000605298 | MIA2 |
| NONHSAT204279.1 | SERF1B |
| NONHSAT212135.1 | PHKG1 |
| ENST00000604992 | GOLGA4 |
| NONHSAT255073.1 | RB1CC1 |
| NONHSAT233811.1 | NEK9 |
| ENST00000621884 | MAPK8IP3 |
| NR_149152.1 | TRPM7 |
| NONHSAT179754.1 | AC011462.1 |
| NONHSAT195845.1 | LRIG1 |
| NONHSAT240597.1 | PNPT1 |
| ENST00000541694 | RHOF |
| NONHSAT236129.1 | ZFHX3 |
| NONHSAT176458.1 | CD79B |
| NONHSAT086726.2 | GTPBP1 |
| NONHSAT069896.2 | LBH |
| ENST00000452809 | ASH1L |
| ENST00000525233 | FAR1 |
| ENST00000501068 | AC092718.8 |
| NONHSAT247227.1 | CTBP1 |
| ENST00000587762 | ZSWIM4 |
| NONHSAT172827.1 | SH2B1 |
| NONHSAT245361.1 | EMC3 |
| ENST00000560518 | MYO5C |
| NONHSAT237656.1 | WDR81 |
| NR_036641.1 | PDGFC |
| ENST00000533924 | NLRP6 |
| ENST00000443093 | AC116366.3 |
| NONHSAT245634.1 | PPP4R2 |
| XR_002957424.1 | LTA4H |
| XR_001749274.2 | LTA4H |
| NONHSAT165394.1 | RPL21 |
| NONHSAT053824.2 | BRCA1 |
| NONHSAT237917.1 | BRCA1 |
| ENST00000557373 | BAZ1A |
| NR_033418.1 | ZSCAN5A |
| ENST00000587620 | ZSCAN5A |
| NONHSAT099604.2 | LRP2BP |
| ENST00000629576 | AC006033.2 |
| NONHSAT253292.1 | AC006033.2 |
| NONHSAT226185.1 | EFCAB2 |
| NONHSAT188509.1 | AL121900.2 |
| NONHSAT237694.1 | RNASEK-C17orf49 |
| NONHSAT176458.1 | AC127029.3 |
| ENST00000623966 | MED26 |
| ENST00000333487 | SREBF2 |
| NONHSAT237332.1 | SUPT4H1 |
| NONHSAT251954.1 | HIVEP1 |
| ENST00000602718 | NDUFA6 |
| NR_126534.1 | AC064801.1 |
| NONHSAT175358.1 | LUC7L3 |
| NONHSAT221505.1 | DPP7 |
| ENST00000628412 | XPC |
| NONHSAT176262.1 | FMNL1 |
| NONHSAT254688.1 | INSIG1 |
| ENST00000584934 | PTP4A1 |
| ENST00000641267 | DENND4B |
| NONHSAT122650.2 | AC004917.1 |
| NONHSAT122651.2 | AC004917.1 |
| ENST00000651902 | AC004917.1 |
| NONHSAT068351.2 | ERVK3-1 |
| NR_033418.1 | ZNF582 |
| NONHSAT155975.1 | PDZD7 |
| NONHSAT237694.1 | ALOX12 |
| ENST00000603199 | XPO1 |
| NONHSAT097602.2 | AC018797.3 |
| ENST00000563278 | SIN3A |
| NONHSAT155975.1 | LZTS2 |
| NONHSAT060631.2 | MYDGF |
| NONHSAT099604.2 | ANKRD37 |
| NONHSAT236913.1 | SPG7 |
| NONHSAT236909.1 | SPG7 |
| NONHSAT060631.2 | TNFAIP8L1 |
| ENST00000601296 | ABHD16B |
| NONHSAT000329.2 | SSU72 |
| ENST00000454613 | MATN1 |
| ENST00000414532 | MATN1 |
| NONHSAT175841.1 | P2RX5 |
| ENST00000602306 | TARBP2 |
| ENST00000569912 | FAM83F |
| ENST00000607978 | RABGEF1 |
| NONHSAT256121.1 | VPS13B |
| NONHSAT156933.1 | ZNF487 |
| NONHSAT248402.1 | THAP9 |
| NONHSAT235609.1 | ABHD2 |
| NONHSAT033737.2 | CDADC1 |
| ENST00000641267 | GATAD2B |
| NONHSAT253549.1 | AC000120.2 |
| ENST00000611877 | CLTC |
| NONHSAT245361.1 | PRRT3 |
| NR_138604.1 | MCRIP2 |
| NONHSAT002770.2 | SLC2A1 |
| NONHSAT234271.1 | FBXO34 |
| ENST00000586376 | FMNL1 |
| NONHSAT173634.1 | C16orf45 |
| NONHSAT188509.1 | DTD1 |
| NONHSAT229620.1 | TMEM9B |
| ENST00000608684 | DNAJB4 |
| NONHSAT086678.2 | TPTEP2-CSNK1E |
| ENST00000608266 | LUC7L2 |
| XR_936967.2 | CABLES2 |
| NONHSAT243420.1 | ZNFX1 |
| ENST00000608450 | PURB |
| NONHSAT237281.1 | ZNF652 |

Table S6. ROC curve analysis of NT-proBNP and lncRNAs.

| ROC characteristics | NT-proBNP | CHST11 | AP000873.3 | MIR29B2CHG | CR381653.1 | FP236383.2 | DLEU2 |
| --- | --- | --- | --- | --- | --- | --- | --- |
| Area under the ROC curve (AUC) | 0.844 | 0.84 | 0.613 | 0.732 | 0.744 | 0.699 | 0.551 |
| Standard Error | 0.0465 | 0.0444 | 0.059 | 0.0497 | 0.0494 | 0.0573 | 0.0668 |
| 95% Confidence interval | 0.736 to 0.921 | 0.754 to 0.906 | 0.507 to 0.713 | 0.635 to 0.815 | 0.647 to 0.826 | 0.592 to 0.793 | 0.436 to 0.663 |
| z statistic | 7.391 | 7.663 | 1.925 | 4.672 | 4.932 | 3.482 | 0.767 |
| Significance level P (Area=0.5) | <0.0001 | <0.0001 | 0.0542 | <0.0001 | <0.0001 | 0.0005 | 0.4429 |
